# Antibody-dependent enhancement of dengue virus infection inhibits RLR-mediated Type-I IFN-independent signalling through upregulation of cellular autophagy

**DOI:** 10.1038/srep22303

**Published:** 2016-02-29

**Authors:** Xinwei Huang, Yaofei Yue, Duo Li, Yujiao Zhao, Lijuan Qiu, Junying Chen, Yue Pan, Juemin Xi, Xiaodan Wang, Qiangming Sun, Qihan Li

**Affiliations:** 1 Institute of Medical Biology, Chinese Academy of Medical Sciences, and Peking Union Medical College, Kunming 650118, PR China; 2 Yunnan Key Laboratory of Vaccine Research & Development on Severe Infectious Diseases, Kunming 650118, PR China; 3 Key Laboratory of The Second Affiliated Hospital of Kuming Medical College, Kunming 650101, PR China

## Abstract

Antibody dependent enhancement (ADE) of dengue virus (DENV) infection is identified as the main risk factor of severe Dengue diseases. Through opsonization by subneutralizing or non-neutralizing antibodies, DENV infection suppresses innate cell immunity to facilitate viral replication. However, it is largely unknown whether suppression of type-I IFN is necessary for a successful ADE infection. Here, we report that both DENV and DENV-ADE infection induce an early ISG (NOS2) expression through RLR-MAVS signalling axis independent of the IFNs signaling. Besides, DENV-ADE suppress this early antiviral response through increased autophagy formation rather than induction of IL-10 secretion. The early induced autophagic proteins ATG5-ATG12 participate in suppression of MAVS mediated ISGs induction. Our findings suggest a mechanism for DENV to evade the early antiviral response before IFN signalling activation. Altogether, these results add knowledge about the complexity of ADE infection and contribute further to research on therapeutic strategies.

Dengue virus (DENV) is a mosquito-borne virus that causes dramatic public health issues in more than 100 countries, particularly in Asia and Latin America. It is estimated that more than 50 million people are infected by DENV annually^1^. The geographic expansion of the vector, the *Aedes aegypti* mosquito, contributes to a continuous increase in the incidence and severity of the disease^2^.

There are four serotypes of DENV (DEVN 1–4), and each of them could cause a spectrum of outcomes from subclinical to death^3^. Moreover, secondary heterotypic infection or waning immunity of infants born to mothers infected by DENV has been observed to significantly increase the likelihood of acquiring severe disease^4^. Moreover, antibody (Ab)-dependent enhancement (ADE) has been thought to be involved in the immunopathogenesis of severe dengue forms, including dengue haemorrhagic fever (DHF) and dengue shock syndrome (DSS). It has been hypothesized that the preexisting heterotypic antibodies form a complex with the virus, via Fc receptors in the target cells, to facilitate the infection of target cells, including monocytes, macrophages and mature DCs^5^,^6^. Many earlier *in vitro* studies have reproduced an enhanced infection of Fc-receptor bearing cells resembling that of DHF/DSS patients^7^,^8^. In addition, passively transferring DENV-specific monoclonal antibodies into an animal model resulted in a notable clinical manifestation and viraemia^9^,^10^. These findings suggest that subneutralizing antibodies are sufficient to induce DHF/DSS in spite of aberrant cellular immunity, which allows exploration of the pathogenesis of severe dengue disease in a culture system.

Given that elevated viraemia is normally accompanied by a high concentration of proinflammatory and immunomodulatory cytokines^11^, it is therefore necessary to understand the connections between the DENV-Ab complex and those cytokines. A previous study using the THP-1 cell line found that DENV-ADE infection could suppress the expression of IL-12, IFN-γ and TNF-α, while stimulating the expression of the anti-inflammatory cytokines IL-6 and IL-10^12^. It was then proposed that DENV-ADE specifically modulated IL-10 production to suppress type I IFN signalling, as well as upregulating dihydroxyacetone kinase (DAK) and autophagy-related 5 (ATG5) to restrain IFN-α/β production^13^. Another study using human macrophages also revealed a similar function of IL-6, but not IL-10, that was regulated by ADE^14^. All these results suggest the importance of anti-inflammatory cytokines in the IFN antiviral pathway, especially IL-10^15^. However, it is so far unclear whether the induction of IL-10 or IL-6 could directly increase cellular viral replication or whether they are only the byproducts of DENV-ADE infection. In addition, the phenomenon that DENV-ADE infection suppresses the secretion of type I IFN was not found in any other *in vitro* studies using human primary monocytes^7^,^16^. Therefore, it is reasonable to postulate a more pervasive mechanism in DENV-ADE infection, which does not rely on the suppression of IFNα/β or increased IL-10/IL-6.

In this study, we used the IFN-deficient monocytic cell line K562 to show that ADE effects are independent of the suppression of type I IFN. Meanwhile, both DENV and DENV-ADE infection induced direct expression of NOS2 through activation of the RIG-I/MDA-5-MAVS signalling axis. We further report that DENV-ADE induced higher expression of autophagy-related proteins (ATG5-ATG12) and elevated autophagosome formation to facilitate viral replication. This supplies a new strategy for DENV-ADE to contend with innate cell immunity in the context of extensive IFN antagonism.

## Results

### The enhancement activity of DENV-ADE infection is dependent on the final concentration of anti-PrM antibody

**Figure 1:**
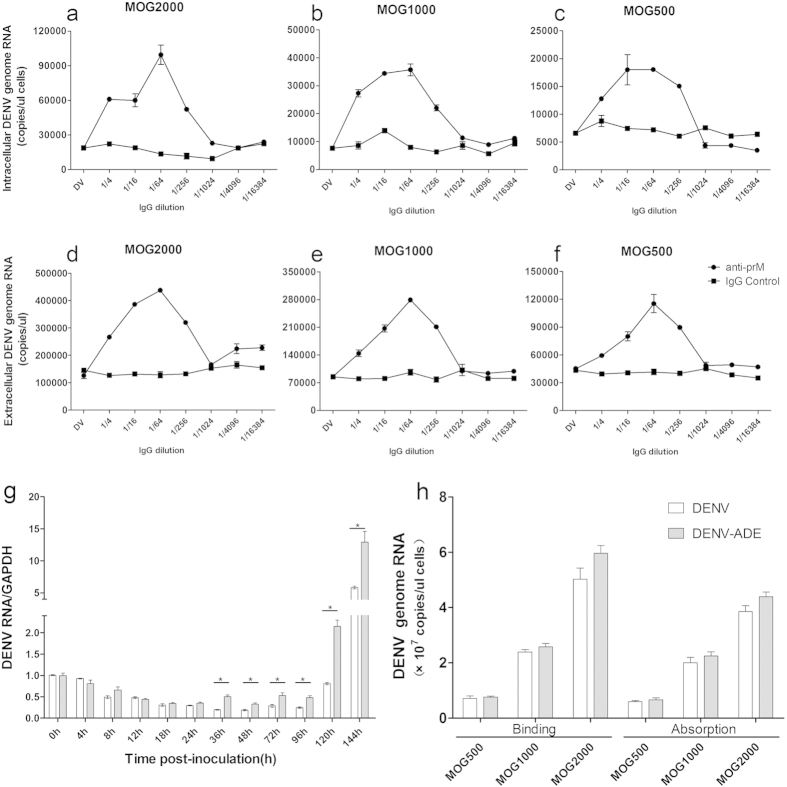
DENV-ADE infection increased virus replication through an intrinsic pathway. K562 cells were infected with DENV3 at a MOG of 2000, 1000 and 500 complexed with 4-fold dilutions of anti-prM mAb or mock IgG. At 48 h post-infection, cells (**a–c**) and supernatants (**d–f**) were collected for total RNA extraction; the DENV genome RNA was quantified (copies/μl) using qRT-PCR. N = 3; error bars show the means ± SEM. (**g**) Kinetics of DENV3 intracellular RNA accumulation analysed by relative qRT-PCR. K562 cells were infected with DENV3 alone or DENV3-Ab complex at a peak enhancement dilution (1/64) at MOG = 1000 and harvested at the indicated times post-inoculation. An equal amount of total RNA from each sample was subjected to relative quantitation and GAPDH was used as an internal control. N = 2. (**h**) Binding and internalization results of DENV3 and DENV3-Ab complex on K562 cells by qRT-PCR. N = 3; error bars show the means ± SEM.

Distinct types of monocytes such as THP-1, U937 and K562 have been extensively used to explore the mechanisms of dengue virus ADE infection using prototype dengue viral strains^17^. For simulation of a natural infection, we chose low-passage DENV3 isolates and the anti-DENV2 PrM antibody to establish an *in vitro* model of DENV-ADE infection in K562 cells. ELISA and neutralization assays indicated that the anti-PrM antibody is capable of binding to the DENV3 isolate but exhibits no neutralization activity (Fig. S1). As shown in Fig. 1a–f, serial dilutions of PrM antibodies promoted the synthesis of the intracellular DENV genome and increased the total genome-containing particles (GCP) in supernatants corresponding to different MOG (multiplicity of genome containing particles) sets (MOG = 2000, 1000, and 500). These results indicate that the increased DENV GCP in ADE infection may result from elevated intracellular virus RNA accumulation or synthesis. Interestingly, the enhancement activity of DENV-ADE infection is dependent on the final antibody concentration rather than the DENV/antibody ratio in all three MOG sets, as they all exhibited the same peak enhancement at an antibody dilution of 1/64. This phenomenon was concordant with the results found in FcγR-expressing BHK cells^18^.

### Increased virus uptake insufficiently accounted for the elevated virus production in ADE infection

Although increased viral uptake was suggested to be the major component of ADE infection, i.e., the extrinsic ADE, the binding and absorption mechanism during ADE infection has not been fully examined. To determine whether the enhanced viral production results from increased viral uptake, we first examined the intracellular viral RNA accumulation in DENV/DENV-ADE infected K562 cells at distinct time points post-inoculation by relative quantification. As shown in Fig. 1g, a significant difference in the viral RNA levels between ADE and direct infection was observed from 36 h post infection. Inconsistent with the previous studies, the ADE infection does not facilitate viral uptake during infection, owing to the similar cellular viral RNA levels at first 24 h post-infection. To further address this issue, we used a more precise assay to evaluate the viral binding and absorption as described in a previous study^19^. We found that either ADE infection or direct infection exhibited similar levels of virus binding and absorption at all 3 sets of MOG (Fig. 1h). Collectively, our results suggested an intrinsic pathway for enhancement of DENV infection mediated by antibodies.

### DENV-ADE infection suppresses NOS2 expression and activity to facilitate viral RNA synthesis

**Figure 2:**
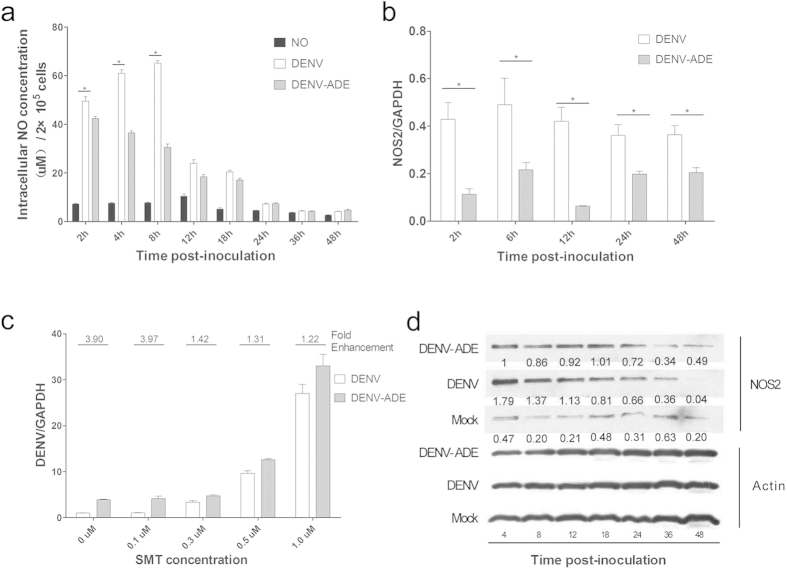
The suppression of NOS2 expression and subsequent NO production in DENV-ADE infected K562 cells. (**a**) K562 cells were infected with W/WT DENV or DENV-antibody complex at a MOG of 1000, and at the indicated time points, cellular NO production was quantified by the Griess reaction. N = 3; error bars show the means ± SEM. The results from each group were compared using Student’s t test. **P < 0.01; *P < 0.05. (**b,d**) The expression of NOS2 at the indicated time points was determined at the gene transcription level by qRT-PCR (**b**) and at the protein level by western blotting (**d**). N = 3; error bars show the means ± SEM. (**c**) K562 cells were pretreated with different concentrations of SMT 1 h before DENV-3 or DENV-ADE infection, and cellular DENV genome RNA was extracted 48 h post-infection and quantified using qRT-PCR, with GAPDH as an internal control. N = 3; error bars show the means ± SEM.

As an effector of the innate immune system, inducible nitric oxide synthase (NOS2)-derived nitric oxide (NO) can evoke a set of rapid host responses to pathogens. The lower concentration of serum NO was shown to correlate with severe dengue symptoms^20^. Moreover, *in-vitro* studies reported that NO inhibited DENV RNA synthesis through attenuation of the RNA-dependent RNA polymerase (RdRp) activity of DENV-NS5^21^. Due to the deficiency of type-I IFN genes in K562 cells^22^ (determined by IFNα/β dot-blot assay, as shown in Fig. S2), it is interesting to determine whether the intrinsic DENV-ADE pathway decreases NO generation for virus replication. Thus, we first evaluated the NO concentration in DENV/DENV-ADE infected cells at different time points. The NO level first exhibited a dramatic increase during the early infection, and direct DENV infection induced a significantly higher level of NO than DENV-ADE infection within 4–8 h post-inoculation (Fig. 2a). Subsequent detection of NOS2 expression changes revealed that DENV-ADE infection suppress NOS2 transcription with during the whole process of infection but suppress protein level only at very early time points (4–8 hpi), shown in Fig. 2b,d. The above results suggested that DENV-ADE infection could induced an early suppression of NOS2 translation, which may help virus avoid early detection by cell innate immune system. To investigate whether the enhanced viral production in DENV-ADE infected cells is the result of NOS2 functional suppression, we used cells pre-treated with the NOS2 specific inhibitor SMT at the concentrations of 0.1, 0.3, 0.5, and 1.0 μM before infection with DENV or the DENV-Ab complex. Cell viability was not significantly affected by the SMT treatment (Fig. S3). As shown in Fig. 2c, the relative quantitation of the cellular viral RNA level suggested that SMT could promote the accumulation of cellular viral RNA in a dose-dependent manner, as well as decreased fold enhancement of DENV-ADE infection. Altogether, these results indicated that in the absence of type I IFN, DENV-ADE infection could impair the innate immune response through diminished NOS2 function.

### Suppression of RIG-I and MDA-5 signalling in DENV-ADE-infected K562 cells

**Figure 3:**
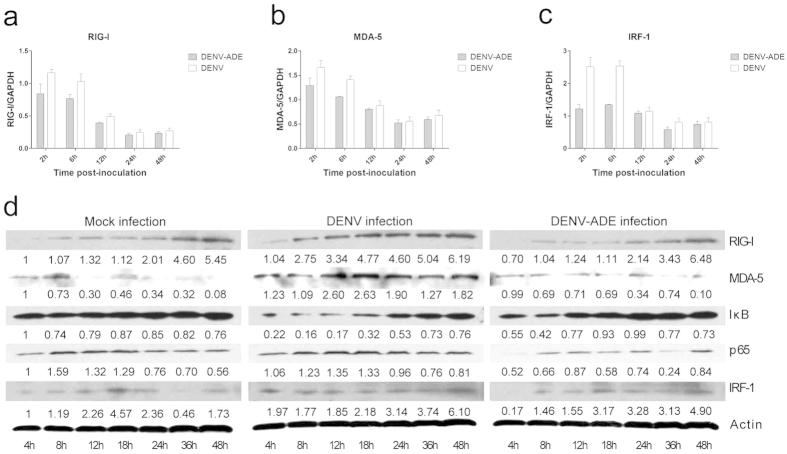
Suppression of the RIG-I and MDA-5 signalling pathways in DENV-ADE infected K562. K562 cells were infected with DENV alone or DENV-antibody complex or were mock infected. (**a–c**) The relative quantification of total cellular RNA was performed using qRT-PCR with GAPDH as an internal control. The transcription level of each gene in cells before infection was designated as a basal transcription level and normalized as 1. N = 3; error bars show the means ± SEM. Student’s t test was performed for each group. **P < 0.01; *P < 0.05. (**d**) Cell lysates post infection were subjected to sodium dodecyl sulfate polyacrylamide gel electrophoresis and stained with specific antibodies against RIG-I, MDA-5, iκ-B, NF-κB p65 and IRF-1 at the indicated time points. N = 3.

RIG-I (retinoic acid-inducible gene-I)-like receptors (RLRs), including RIG-I and MDA-5 (melanoma differentiation-associated gene 5), can recognize viral RNA and transmit an antiviral signal from the mitochondria antiviral protein (MAVS) thus inducing type I IFN signalling^23^. In addition, DENV-ADE infection was shown to decrease IFN-β secretion by suppression of MAVS-mediated signalling^24^. We thus asked whether DENV-ADE infection suppresses RIG-I and MDA-5 mediated antiviral signalling in the absence of type I IFN. Therefore, the expression levels of RIG-I and MDA-5, and their downstream signalling proteins (NF-κB, iκB, and IRF-1) extracted from DENV or DENV-ADE infected K562 cells were compared by Western blotting. As shown in Fig. 3, both DENV and DENV-ADE infection could induce activation of RIG-I and MDA-5 signalling. In contrast to DENV infection, DENV-ADE infection induced a lower expression level of RIG-I and MDA-5 and decreased NF-κB activation, which manifested as a degradation of iκ-B and increased expression of NF-κB (subunit p65) (Fig. 3d). The quantification results for mRNA expression also revealed a decline in RIG-I, MDA-5 and IRF-1 transcription in DENV-ADE-infected K562 cells (Fig. 3a–c). Remarkably, in our research, RIG-I and MDA5 signalling was triggered as early as 2 h post-infection, while others reported that IFNs (including IFN α/β/γ) could not be activated even at 6 hours post-infection in the DENV infected THP-1 cells^25^. This implied that MAVS mediated antiviral signalling may be different across different time scales. The early activation of MAVS may participate in innate immunity independent of IFN signalling.

### RLRs signalling suppresses intracellular DENV RNA synthesis through direct induction of NOS2

**Figure 4:**
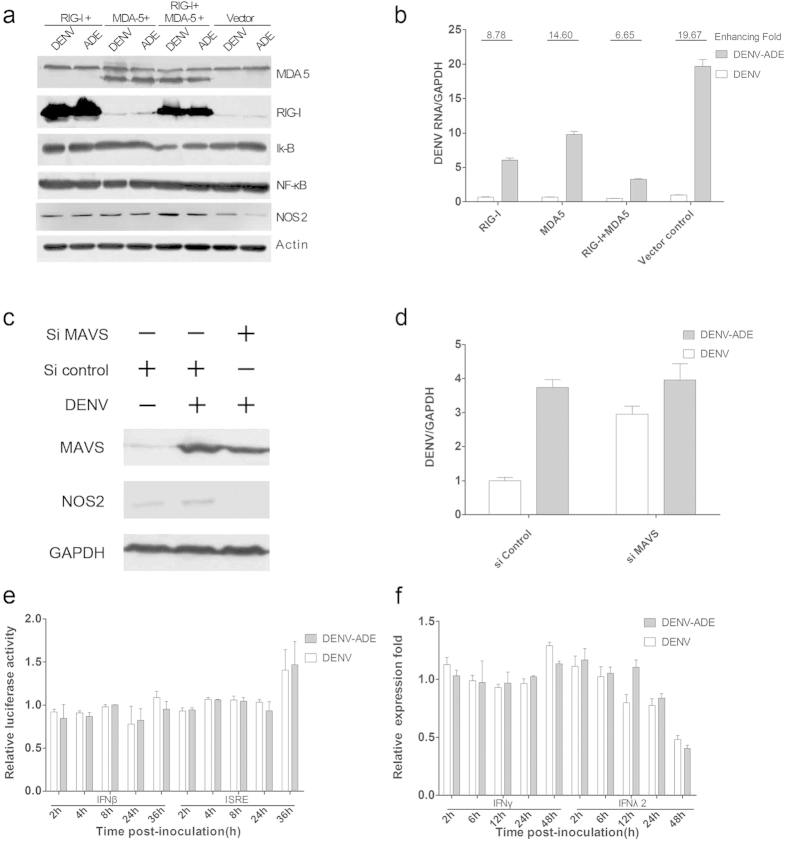
DENV/DENV-ADE-induced RLR activation mediates NOS2 expression independent of IFN signalling. K562 cells was stably transfected with plasmid expressing RIG-I W/WT MDA-5. All the cells were subjected to DENV and DENV-ADE infection. (**a**) Cell lysates were subjected to sodium dodecyl sulfate polyacrylamide gel electrophoresis and stained with specific antibodies against RIG-I, MDA-5, iκ-B, NF-κB and NOS2 6 h post-inoculation. N = 3. (**b**) Cells were harvested for total RNA 48 h post-inoculation; the intracellular viral RNA was detected by qRT-PCR, with GAPDH as an internal control. The fold enhancement was expressed as the ratio of DENV/DENV-ADE. N = 3; error bars show the means ± SEM. K562 cells was transfected with siRNA target MAVS or siRNA control. (**c**) cell lysates were subjected to detection of MAVS and NOS2 4 h post-infection. (**d**) Total cellular RNA was extracted for intracellular viral RNA quantitation 48 h post-inoculation. (**e**) K562 cells were co-transfected with pLR-TK plasmid (internal control) and pGL3 -hIFNβ or pGL3-ISRE plasmid (experimental reporter) and then subjected to DENV or DENV-ADE infection. At the indicated time points post-infection, reporter gene activity was measured by a dual-luciferase reporter assay. N = 3; error bars show the means ± SEM. (**f**) K562 cells were infected with DENV alone, DENV-antibody complex, or mock infected. Cellular RNA was extracted and used for gene expression quantification of IFNγ and IFNλ using qRT-PCR at indicated time points. N = 3; error bars show the means ± SEM.

As an important ISG (interferon-stimulated gene), NOS2 can be activated following stimulation of type I IFNs and IFNγ^26^. Moreover, there are other innate immune pathways including the mitogen-activated protein kinase (MAPK) or NF-κB pathway which are able to activate the transcription of NOS2^27^. Then, we reasoned that the decreased NOS2 expression in DENV-ADE infected K562 cells was due to suppression of RIG-I/MDA-5 signalling. First, overexpression of RIG-I with or without MDA-5 can facilitate the activation of NF-κB complex manifested as a degradation of iκ-B and increased expression of NF-κB (subunit p65) and leading to increased expression of NOS2 (Fig. 4a). Such induction of NOS2 consequently suppresses virus replication both in DENV/DENV-ADE infected cells, as well as by enhancing the ability of DENV-ADE (Fig. 4b). In contrast, knockdown of MAVS downstream of RIG-I/MDA-5 signalling dramatically abolished NOS2 induction both in DENV/DENV-ADE infected cells and the enhanced ability of DENV-ADE (Fig. 4c,d). Nevertheless, it is possible that the NOS2 production is activated by IFN-associated signalling pathways. To test this, we used a dual-luciferase reporter assay to determine the activity of the IFN-β and ISRE reporter in DENV or DENV-ADE infected K562 cells. As shown in Fig. 4e, no activation of IFN-β and ISRE reporters was found in DENV or DENV-ADE infected K562 cells. In addition, DENV or DENV-ADE infection did not induce an increased transcription of IFN-γ and IFN-λ2 (Fig. 4f). Overall, these results suggested that RIG-I/MDA-5 mediated type I IFN-independent pathway activation in DENV/DENV-ADE infection, and DENV-ADE infection depressed such signalling for elevated viral replication.

### Redundant function of IL-10 in DENV-ADE infection of K562

**Figure 5:**
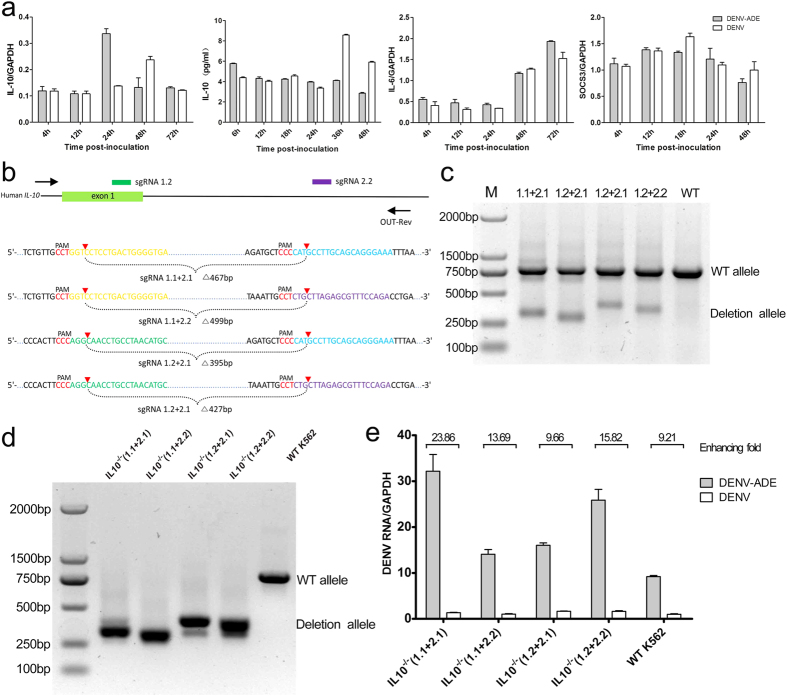
DENV-ADE infection of K562 is independent of IL-10 function (**a**) K562 cells were infected with DENV alone or DENV-antibody complex or were mock infected. Cellular RNA was extracted and use for gene expression quantification of IL-10, IL-6 and SOCS3 using qRT-PCR; the supernatants of cell cultures were tested for IL-10 using an ELISA kit. N = 3; error bars show the means ± SEM. (**b**) Four pairs of sgRNAs were designed to target the human IL-10 loci. Target sequences and PAMs (red) are shown in respective colours, and sites of cleavage by Cas9 are indicated by red triangles. A predicted junction is shown below. (**c**) Cell populations transfected with paired sgRNAs (1.1 + 2.1, 1.1 + 2.2, 1.2 + 2.1, 1.2 + 2.2) were assayed by PCR (Out-Fwd and Out-Rev primers were used), reflecting a deletion of ~270 bp. (**d**) Paired sgRNA transfected cells were clonally isolated and expanded for genotyping analysis of deletions using PCR (using the Out-Fwd and Out-Rev primers) and sequencing. Each isolate of K562 with homozygous deletions was subjected to DENV-ADE/DENV infections. (**e**) Cellular dengue genome RNA was quantified using qRT-PCR with GAPDH as an internal control; the fold enhancement in each K562 isolate was expressed as a ratio of DENV/DENV-ADE. N = 3; error bars show the means ± SEM.

The elevation of IL-10 is implicated in the pathogenesis of severe dengue diseases based on the clinical observations^11^,^28^. IL-10 has multiple cellular functions, including immunosuppressive effect exerted through the SOCS system and JAK-STAT pathway. However, the precise role of IL-10 in DENV-ADE infection, especially in a single cell culture system, remains unknown. In this study, we detected the IL-10 expression in DENV or DENV-ADE infected K562 cells at both the transcriptional and protein levels. As shown in Fig. 5a, no significant increase in IL-10 or IL-6 expression was found in the DENV-ADE infected K562 cells, as well as for SOCS3. To further confirm the role of IL-10 in DENV-ADE infection, we generated four mutant K562 strains carrying homozygous deletions of IL-10 using the CRISPR-Cas9 approach (Fig. 5b,c). All four strains expressed no reduction in the fold enhancement of DENV-ADE compared with the wild-type K562 cell line, as shown in Fig. 5d,e. These data indicated that the marked increase of viral production in DENV-ADE infected K562 was independent of IL-10.

### Increased Autophagy in DENV-ADE-infected K562 cells promotes viral RNA replication

**Figure 6:**
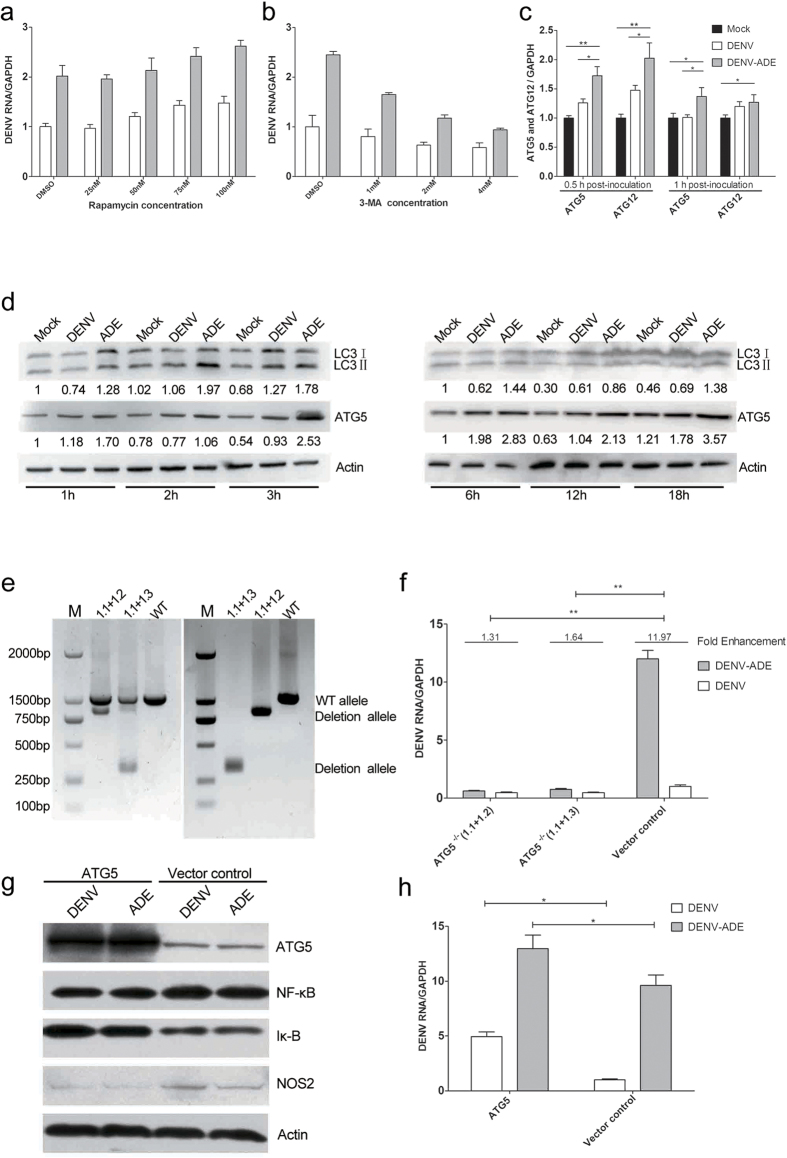
DENV-ADE infection of K562 induces greater autophagy to facilitate viral production. K562 cells pretreated with different concentrations of rapamycin (**a**) or 3-MA (**b**) 1 h before infection with DENV-3 or DENV-ADE. Forty-eight hours post-infection, intracellular DENV RNA was extracted and quantified using qRT-PCR. N = 3; error bars show the means ± SEM. (**c**) K562 cells were infected with DENV alone, DENV-antibody complex, or mock. At both 0.5 h and 1 h post-inoculation, cellular RNA was extracted and used for gene expression quantification of ATG5 and ATG12 using qRT-PCR. N = 3; error bars show the means ± SEM, the results from each group were compared using Student’s t test. **P < 0.01; *P < 0.05. (**d**) K562 cells were infected with DENV alone, DENV-antibody complex, or mock. Cell lysates were subjected to detection of LC3 I/II, ATG5 and β-actin at the indicated time points. N > 3. (**e**) K562 cells transfected with paired sgRNA (1.1 + 1.2, 1.1 + 2.3) targeting the *ATG5* locus were assayed by PCR (using the ATG5-Out-Fwd and ATG5-Out-Rev primers). Clonal isolates carrying a homozygous deletion in the *ATG5* locus was were using PCR and sequencing. (**f**) Each isolate was subjected to DENV-ADE/DENV infection. Intracellular dengue genome RNA was quantified using the qRT-PCR method with GAPDH as an internal control, and the fold enhancement in each K562 isolate was expressed as a ratio of DENV/DENV-ADE. N = 3; error bars show the means ± SEM, the results from each group were compared using Student’s t test. **P < 0.01; *P < 0.05. K562 cells were stably transfected with plasmid expressing ATG5 or empty plasmid (control). All cell lines were subjected to DENV and DENV-ADE infection. (**g**) 4 h post-inoculation, cell lysates were subjected to detection of iκ-B, NF-κB, NOS2 and β-actin. N = 3. (**h**) At 48 h post-inoculation, cells were harvested for total RNA, and the intracellular viral RNA was detected using qRT-PCR with GAPDH as an internal control. N = 3; error bars show the means ± SEM, the results from each group were compared using Student’s t test. **P < 0.01; *P < 0.05.

It was shown that most positive-strand RNA viruses utilize the maturation of autophagosomes into acidic and ultimately degradative compartments to promote their replication^29^. Moreover, the autophagy related proteins ATG5 and ATG12 were implicated in RLR signalling through interaction with MAVS^30^,^31^. Therefore, it is important to determine whether the elevated viral production in DENV-ADE infected K562 is attributed to an increased cellular autophagy process. First, we pretreated K562 cells with either an accelerator (rapamycin) or an inhibitor (3-MA) of autophagy before infecting them with DENV or DENV-antibody complex. As shown in Fig. 6a, intracellular viral RNA synthesis was increased by rapamycin in a dose dependent manner. Meanwhile, the decreased DENV viral RNA synthesis was 3-MA dosage dependent (Fig. 6b). Moreover, DENV-ADE infection induced higher ATG5 and ATG12 transcription at very early stage (0.5 and 1 hours post-infection) compared to the DENV direct infection (Fig. 6c). When we examined the phenomenon at the protein level, an early increased ATG5 expression and LC3II formation was observed in DENV-ADE infected cells compared to DENV infected cells (Fig. 6d). To further confirm the role of the autophagy process in DENV infection, ATG5 knockout K562 cells were generated using the CRISPR-Cas9 method as described earlier (Fig. 6e). As shown in Fig. 6f, a significant decrease in intracellular viral RNA synthesis was detected in two mutant strains of K562 carrying homozygous deletions of ATG5 after DENV and DENV-ADE infection. Remarkably, the enhanced activity was abrogated following ATG5 complete knockout. Overexpression of ATG5 impaired NF-κB activation following NOS2 expression and eventually led to increased intracellular viral RNA replication in both DENV and DENV-ADE infected cells (Fig. 6g,h). Our results implied that DENV-ADE infection in K562 utilized the elevation of autophagy for maximum viral production. There is also a possibility that DENV-ADE infection could utilize the elevated ATG5 and ATG12 to antagonize the RLRs signalling pathway.

## Discussion

K562 is a widely used cell line to test the ADE activity of DENV antibodies. However, it is not clear whether the enhanced cellular viral production is attributed to extrinsic ADE rather than alterations in the antiviral signalling pathways. When the comparison of progeny virions per cell was applied to yield a similar percentage of infected cells, there was no significant increase in viral output between normal infection and ADE infection in K562 cells^8^. Nevertheless, it may be inadequate to draw the conclusion that K562 cells could only express extrinsic ADE infection, especially when the cell infection rate was measured by immunostaining techniques in which unstained cells may also carry the viral RNA^32^. In this study, we chose a more sensitive method. qPCR was used to detect viral RNA synthesis post-inoculation. As shown in Fig. 1, similar viral RNA levels were found in both DENV infected and DENV-ADE infected cell in the first few hours post infection, while they started to behave differently in terms of viral RNA levels 36 h post infection. The phenomenon exhibited a representative DENV intrinsic ADE infection. Moreover, we observed that the antibody enhancement did not significantly promote virion internalization, which also implied an intrinsic ADE pathway.

Upon DENV infection, cellular RLRs are activated to elicit anti-viral responses depending on the induction of type I IFN and proinflammatory cytokines. The binding of type I IFN with its receptor then activates multi-subsets of ISGs through JAK-STAT signalling which amplifies and sustains the initial antiviral responses^33^. ISGs can also be activated in IFN independent pathways^34^. In this study, we provide evidence that DENV/DENV-ADE infection of monocytes induces early production of ISG (NOS2) through the RIG-I/MDA-5-MAVS-NF-κB/IRF-1 signalling axis independent of the IFN pathway. This is consistent with the recent finding that 5′-triphosphorrylated RNA (5′pppRNA) restricts DENV infection by a RIG-I mediated type I IFN-independent pathway^35^. However, more than one mechanism of RLR-induced ISG expression may operate in virus-infected cells based on the MAVS localization. Peroxisome-localized MAVS are activated early during the induction of ISGs whereas mitochondrial MAVS promote IFN secretion and ISG expression with delayed kinetics, at least in fibroblasts^36^. However, whether the early induction of NOS2 through RLR in this study comes from peroxisomal MAVS remains to be confirmed. After all, the early induction of ISGs may be due to virus entry such as WNV in a IFN-, TLR-, and RIG-I independent manner^37^. Nevertheless, alternative ISGs induction would enable cells to control pathogens that disrupt the expression of IFNs or antagonize IFN signalling, which include DENV, WNV, and VSV. In addition, the rapid induction of ISGs before IFN transcription would help to orchestrate a robust innate cell immunity that cooperates with delayed IFN signalling. Meanwhile, this also suggests the importance of suppressing early ISG activation in a successful DENV-ADE infection. It was well reviewed that the antiviral response was triggered during the early stage (binding, entry) of the viral life cycle. Investigation of the difference in gene expression pattern within first hours post-infection would help to understand the phenomenon of intrinsic ADE^38^.

In DENV infection with enhancing antibodies, ligation of FcγRs was shown to induce ISGs through the ITAM-Syk-STAT1 signalling axis independent of autocrine or paracrine IFN activity^25^,^39^. This means that the antibody-mediated viral entry would have no benefit for virus production. Previous study suggested that inhibition of the innate immunity with coligation of LILRB1 would ensure a successful ADE infection^25^. However, the LILRB1 polymorphisms were not correlated with dengue shock syndrome in genome-wide association analysis^40^. In addition, recent studies demonstrated that ADE infection induced inflammatory cytokine expression through the Syk-ERK signalling axis^41^. Remarkably, inhibition of Syk activation reduced ADE-induced inflammatory cytokine expression while barely impairing virus production^41^. Therefore, it is reasonable to think that enhancing antibodies contribute to deadly pathology either through Syk-induced massive and aberrant production of cytokines or through other ways to induce elevated virus production.

There is another cytokine known as IL-10 that is implicated in the pathogenesis of severe dengue diseases. The elevation of serum IL-10 is positively correlated with the development of viraemia and clinical manifestations in many observations^11^,^42^. There is a hypothetical intrinsic pathway that was proposed for either IL-10 or IL-6 in DENV-ADE infection of monocytes and macrophages^17^,^18^. Based on this hypothesis, antibody-mediated increases in the production of IL-10 and downstream SOCS3 were considered to be the origin of elevated viral products^13^. However, the total production of IL-10 was attributed to single nucleotide polymorphisms in its promoter region^7^. Monocytes isolated from homozygous GCC, ACC, and ATA donors exhibit a remarkable difference in IL-10 production but a similar pattern in DENV-ADE infection^7^. Moreover, a late peak of IL-10 production after viraemia at defervesce did not support a strong role for IL-10 in DENV-ADE infection^43^. Therefore, the function of IL-10 in the pathogenesis of DHF/DSS appears to be more obscure. To determine the role of IL-10 in DENV-ADE infection, 4 distinct strains of IL-10^−/−^ knockout K562 cell lines were constructed based on the CRISPR-Cas9n double nicking approach. Unexpectedly, all 4 IL-10^−/−^ knockout K562 strains sustained DENV-ADE infection without decreased enhancing activity compared with that found in wild type K562. Our results indicate that IL-10 production has little effect on DENV-ADE infection of K562 cells. In addition, the well-described immunosuppressive function of IL-10 may be mediated through bystander effects, which means the IL-10 inhibits the DENV-specific T cell response to contribute to the pathogenesis of acute dengue infections^44^. Furthermore, a recent study proved that DENV infection or attachment could increase the expression of IL-10 in THP-1 cells, and modulate the influence of IL-10 expression on viral production^45^. This is not surprising, because the function of IL-10 may be mediated through the suppression of latter activated IFN signalling, which consequently explains why IL-10 appears redundant in the DENV infected K562 cells or peripheral blood mononuclear cells which have a lower IFNα/β expression level^7^,^17^,^46^. Combined with our data, it is reasonable to postulate that the DENV-ADE infection may suppress the cellular antiviral response by suppressing not only early induction of ISGs but also the later IFN signalling with IL-10 or IL-6. Furthermore, considering the function of DENV in IFN antagonism^47^,^48^,^49^,^50^, the early suppression of ISG induction could be more important for a successful ADE infection.

Autophagy is a fundamental cellular homeostatic process that digests interior portions of the cell to recycle nutrients or remove unwanted cytoplasmic components. The direct protection against intracellular pathogens by autophagy is dependent on a lysosomal degradation pathway. However, some pathogens including DENV were able to subvert autophagy for their benefit^51^. Previous studies suggested that the DENV virus utilized autophagic machinery for viral replication in liver cell lines^52^ and AG129 mice^53^ but not in the monocytic cell line U937^54^. Based on these studies, it was hypothesized that the double-membrane autophagosome could be a scaffold for viral RNA replication^55^,^56^. In addition, autophagy-induced anti-apoptotic activity could prevent cell death before viral assembly^57^. However, autophagy can engage in the regulation of anti-viral signalling with autophagy initiator proteins, because the deletion of ATG5 or ATG12 could induce the amplification of MAVS mediated signalling^58^. Moreover, ATG5-ATG12 conjugation is able to block the type-I IFN pathway by association with RIG-I and MAVS^30^. In this study, we demonstrated that the wild-type DENV3 required autophagosome formation for maximum virus replication, and DENV-ADE specifically upregulated autophagosome formation and ATG5 expression at very early stage which suppresses MAVS mediated Type I IFN-independent immunity. This is a new strategy for DENV-ADE to contend with innate cell immunity in the context of extensive IFN antagonism. However, it was still unknown that why ADE could induce more autophagy formation. In DENV-ADE infection, FcγR-mediated phagocytosis was proposed as an important entry mechanism based on research of another closely related virus WNV^38^. It has been demonstrated that components of autophagy, like LC3, can also participate in the FcγR mediated phagocytosis, a process termed as LAP (LC3 associated phagocytosis)^59^. However, a recent study reported that phagocytosis also activated the autophagy pathway, which not merely employed certain autophagy proteins^60^. The elevated autophagy process during phagocytosis may participate in lysosomal degradation of the non-self material and cellular immunoregulation. Therefore, it may be assumed that ADE infection induced phagocytosis of virus-antibodies complex also activated autophagy process simultaneously. Nevertheless, further identification of the entry process in ADE will help to explained the link between ADE and autophagy.

In conclusion, we demonstrated an additional mechanism involved in DENV-ADE infection, in which cross-reactive antibodies mediated infection by inducing autophagy related proteins to suppress the innate immunity mediated by MAVS directed ISGs activation. Our findings highlight autophagy inhibitors as superior candidate targets compared to conventional antiviral stimuli with IFNs. Because dengue ADE is broadly relevant to the pathogenesis of viral infection and has hindered dengue vaccine development, our study will help to understand how dengue ADE mediates viral infection, increase our knowledge about the complexity of the host innate response to viral infection and help with vaccine and novel therapy development.

## Materials and Methods

### Cell lines and virus

Human myelogenous leukaemia K562 cells and *Aedes albopictus* mosquito (C6/36) cells were maintained in RPMI-1640 medium (1640, GIBCO) supplemented with 10% foetal bovine serum (FBS), 100U/ml penicillin and 100 μg/ml streptomycin.

The wild type dengue virus strains (DENV3) used in this study were isolated from the blood sample of severe dengue patients at Xishuangbanna Dai Autonomous Prefecture People’s Hospital in 2013, and propagated in C6/36 cells. Written consent was obtained from each participant dengue patients. The study protocol was approved by the institutional ethics committee (Institute of Medical Biology, Chinese Academy of Medical Sciences, and Peking Union Medical College) and was in accordance with the Declaration of Helsinki for Human Research of 1974 (last modified in 2000). These DENV3 isolates were confirmed by genome sequencing (gb: KR296743). Briefly, a monolayer of C6/36 cells was infected at 1 multiplicity of infection (MOI 1) at 28 °C. Five days after infection, the medium containing virus particles were harvested, cleared from cell debris by centrifugation at a speed of 4000 rpm for 10 min, aliquoted and stored at −80 °C. DENV3 was used after three passages in C6/36 cells.

### Viral RNA copy number titration by quantitative PCR analysis

The DENV3 viral RNA standard was prepared by amplifying a 464-bp fragment of DENV3 strain (GI:163644368) with primers 5′-ACAGTTTCGACTCGG-3′ (corresponding to genome position 29 to 43) and 5′-CTTTCATTCTTCCCC-3′ (corresponding to genome position 492 to 478). The resulting product was ligated into pGEM-T Easy vector (Promega) and transformed in DH5a competent cells (Invitrogen). Minipreps of isolated plasmid DNA were then prepared (Promega). RNA transcripts were generated using the Megascript kit according to the manufacturer’s specifications, and the RNA concentration was determined by UV spectrophotometry. Primers (DV3-qF 5′-AACCGTGTGTCAACTGGATCA-3′ and DV3-qR 5′-TTAATGGCCCCCGACTTCTT-3′) were designed to target the DENV pre-membrane region (prM) within the viral RNA standard that was previously designed. Real-time PCR was conducted using SYBRGreen I Mastermix (Applied Biosystems) on a Bio-Rad CFX96 detection instrument. Amplification conditions were 95 °C for 30 s, and 40 cycles of 95 °C for 5 s, 55 °C for 20 s, and 72 °C for 30 s. Viral genome copy numbers were calculated from a standard curve generated by an *in vitro* transcribed RNA standard.

### Virus infection and ADE model *in vitro*

For the direct infection, K562 cells were incubated with DENV3 at a multiplicity of 500, 1000 or 2000 genome-containing particles per cell (MOG). The number of genome-containing particles (GCP) was measured by quantitative PCR analysis of viral genome RNA.

The ADE assays were performed as described by others, using Anti-Dengue Virus prM glycoprotein monoclonal antibody (AB41473, ABCAM). Briefly, anti-prM monoclonal antibodies were serially diluted from 1:4 to 1:16384 in a volume of 50 μl. Viruses, at a multiplicity of 500, 1000, or 2000 MOG, were mixed with the antibody dilutions for 1 h at 37 °C with 5% CO_2_ to allow the formation of an immune complex. The DENV-immune complex was then added into 2 × 10^5^ cells in 24-well plates and incubated for 2 h at 37 °C with 5% CO_2_. The cells were washed twice with PBS to remove any remaining DENV-immune complex and suspended in 1 ml of maintenance medium per well for an additional time.

### Quantitation of gene expression levels by real time RT-PCR

qRT-PCR was used to investigate the relative levels of gene expression. Briefly, K562 cellular RNA was extracted using the Promega SV RNA isolation kit and then subjected to reverse transcription with a first-strand cDNA synthesis kit before amplification by qRT-PCR. The primers for NOS2, RIG-I, MDA-5, NF-κB, IRF-1, IL-10, IL-6, SOCS3, ATG5, ATG12 and GAPDH are shown in Table S1. Real-time PCR was conducted using SYBRGreen I Mastermix (Applied Biosystems) with a Bio-Rad CFX96 detection instrument.

### Binding and internalization assay

Measurement of the number of bound or internalized genome-containing particles per cell was performed as described previously^8^,^19^. Briefly, virus or virus-antibody complex was incubated with 2 × 10^5^ K562 cells at MOG 500, 1000, or 2000 for 1 h at 4 °C. For the binding assay, cells were washed three times with ice-cold PBS containing MgCl_2_ and CaCl_2_ (Life Technologies) to remove unbound viruses. Then, the viral RNA was extracted from the cells with a QIAamp Viral RNA mini Kit (QIAGEN) and quantified using the previously described qRT-PCR method. For the internalization assay, the cells were washed as before and resuspended in warm RPMI 1640 medium for an additional 45 minutes incubation at 37 °C.Then, the viral RNA was extracted from the cells and quantified using the qRT-PCR method.

### Plasmids

Human full length RIG-I and MDA-5 were amplified from the K562 cDNA library using a standard PCR method and then cloned into pcDNA3.1 and pcDNA3.1-Flag vectors, respectively. For IL-10 or ATG5 knockout plasmid constructions, each paired guide oligo designed to target regions of the IL-10 or ATG5 genome were synthesized for annealing and ligation into the BbsI sites in pSpCas9 (BB). All of the guide oligo sequences (top/bottom) are shown in Table S1. Sequences for all constructs were verified by DNA sequence analysis. Luciferase reporter gene plasmids pGL3 -hIFNβ and pGL3-ISRE were kind gifts of Dr. Erol Fikrig and Dr. Penghua Wang of Yale University.

### Cell transfection, clonal cell line isolation and luciferase assays

Transfection of K562 cells with pcDNA 3.1 vector encoding human RIG-I or MDA-5 was performed with Lipofectamine 2000 (Invitrogen) according to the manufacturers’ protocols. Transfected cells were selected with 0.4 mg/ml neomycin (G418) for two weeks and then further selected by the limiting dilution method. Stable transfected cells were recovered in complete medium without neomycin. For transfection of K562 cells with pSpCas9(BB) encoding the IL-10 or ATG5 target sequence, each pair of plasmids was mixed equally in a total amount of 500 ng and then transfected using Effectene Transfection Reagent (QIAGEN). Two days after transfection, cells were harvested for DNA extraction. Deletion events were confirmed by PCR using primers (IL-10 OUT-Fwd/IL-10 OUT-reverse and ATG5 OUT-Fwd/ATG5 OUT-reverse, shown in Table S1). Clonal IL-10^−/−^ or ATG5^−/−^ knockout cell lines were isolated by serial dilutions and confirmed by PCR sequencing of the IL-10 or ATG5 locus, respectively. For luciferase assays, K562 cells were transfected with pLR-TK plasmid (Renilla Luciferase was used as an internal control) and pGL3-hIFNβ or pGL3-ISRE plasmid (firefly luciferase, experimental reporter) using Lipofectamine 2000. Twenty-four hours post transfection, cells were recovered in fresh media and subjected to DENV or DENV-ADE infection. At the indicated time points post-infection, reporter gene activity was measured by a dual-luciferase reporter assay, according to the manufacturer’s instructions (Promega). The luciferase activity was expressed as the fold activation (relative to the basal level of mock treated cells after normalization with the co-transfected Renilla luciferase activity).

### Chemicals and cytotoxicity

The effects of S-methylisothiourea sulfate (SMT), rapamycin and 3-methyladenine (3-MA) on the viability of K562 cell cultures were assessed by MTT (3-[4,5-dimethylthiazol]-2,5-diphenyltetrazolium bromide, Sigma-Aldrich) assays. Briefly, K562 cells were pretreated with chemicals at various concentrations used in this study. Then, MTT at a final concentration of 5 mg/ml was added to each well 3 h before detection. Purple formazan was quantified at 570 nm using a spectrophotometer. The percentage of cell death was expressed as the ratio of the OD570 of treated sample to the OD570 of untreated sample.

### Quantitative detection of cellular nitric oxide production

K562 cells infected with DENV or DENV-antibody complex or those that were mock-infected were harvested at the indicated time points. Cell lysis was assayed for nitrite content, as determined by the Griess method. Briefly, 0.1 ml of cell lysate was mixed with 0.1 ml of Griess reagent in a 96-well plates, and the absorbance at 550 nm was read 10 min later. The NO_2_^−^ concentration (mM) was determined by reference to a NaNO_2_ standard curve.

### Western blot detection of protein production

DENV3 infected and DENV3-ADE infected K562 cells were lysed, subjected to electrophoresis, and stained with specific monoclonal or polyclonal antibodies as described elsewhere.

### Detection of cytokine production by ELISA

IL-10 production in DENV3 infected or DENV3 ADE infected K562 cell supernatants at distinct time points was measured using a commercial ELISA kit according to the manufacturer’s instructions.

## Additional Information

**How to cite this article**: Huang, X. *et al.* Antibody-dependent enhancement of dengue virus infection inhibits RLR-mediated Type-I IFN-independent signalling through upregulation of cellular autophagy. *Sci. Rep.* **6**, 22303; doi: 10.1038/srep22303 (2016).

## Supplementary Material

Supplementary Information

## References

[b1] Simmons C. P., Farrar J. J., Nguyen v V. & Wills B. Dengue. The New England journal of medicine 366, 1423–1432, doi: 10.1056/NEJMra1110265 (2012). 22494122

[b2] Eisen L. & Lozano-Fuentes S. Use of mapping and spatial and space-time modeling approaches in operational control of Aedes aegypti and dengue. PLoS neglected tropical diseases 3, e411, doi: 10.1371/journal.pntd.0000411 (2009). 19399163 PMC2668799

[b3] Rodenhuis-Zybert I. A., Wilschut J. & Smit J. M. Dengue virus life cycle: viral and host factors modulating infectivity. Cellular and molecular life sciences: CMLS 67, 2773–2786, doi: 10.1007/s00018-010-0357-z (2010). 20372965 PMC11115823

[b4] Kliks S. C., Nimmanitya S., Nisalak A. & Burke D. S. Evidence that maternal dengue antibodies are important in the development of dengue hemorrhagic fever in infants. The American journal of tropical medicine and hygiene 38, 411–419 (1988). 3354774 10.4269/ajtmh.1988.38.411

[b5] Jessie K., Fong M. Y., Devi S., Lam S. K. & Wong K. T. Localization of dengue virus in naturally infected human tissues, by immunohistochemistry and *in situ* hybridization. The Journal of infectious diseases 189, 1411–1418, doi: 10.1086/383043 (2004). 15073678

[b6] Balsitis S. J. *et al.* Tropism of dengue virus in mice and humans defined by viral nonstructural protein 3-specific immunostaining. The American journal of tropical medicine and hygiene 80, 416–424 (2009). 19270292

[b7] Boonnak K., Dambach K. M., Donofrio G. C., Tassaneetrithep B. & Marovich M. A. Cell type specificity and host genetic polymorphisms influence antibody-dependent enhancement of dengue virus infection. Journal of virology 85, 1671–1683, doi: 10.1128/JVI.00220-10 (2011). 21123382 PMC3028884

[b8] Rodenhuis-Zybert I. A. *et al.* Immature dengue virus: a veiled pathogen? PLoS pathogens 6, e1000718, doi: 10.1371/journal.ppat.1000718 (2010). 20062797 PMC2798752

[b9] Balsitis S. J. *et al.* Lethal antibody enhancement of dengue disease in mice is prevented by Fc modification. PLoS pathogens 6, e1000790, doi: 10.1371/journal.ppat.1000790 (2010). 20168989 PMC2820409

[b10] Goncalvez A. P., Engle R. E., St Claire M., Purcell R. H. & Lai C. J. Monoclonal antibody-mediated enhancement of dengue virus infection *in vitro* and *in vivo* and strategies for prevention. Proceedings of the National Academy of Sciences of the United States of America 104, 9422–9427, doi: 10.1073/pnas.0703498104 (2007). 17517625 PMC1868655

[b11] Green S. *et al.* Elevated plasma interleukin-10 levels in acute dengue correlate with disease severity. Journal of medical virology 59, 329–334 (1999). 10502265

[b12] Chareonsirisuthigul T., Kalayanarooj S. & Ubol S. Dengue virus (DENV) antibody-dependent enhancement of infection upregulates the production of anti-inflammatory cytokines, but suppresses anti-DENV free radical and pro-inflammatory cytokine production, in THP-1 cells. The Journal of general virology 88, 365–375, doi: 10.1099/vir.0.82537-0 (2007). 17251552

[b13] Ubol S., Phuklia W., Kalayanarooj S. & Modhiran N. Mechanisms of immune evasion induced by a complex of dengue virus and preexisting enhancing antibodies. The Journal of infectious diseases 201, 923–935, doi: 10.1086/651018 (2010). 20158392

[b14] Rolph M. S., Zaid A., Rulli N. E. & Mahalingam S. Downregulation of interferon-β in antibody-dependent enhancement of dengue viral infections of human macrophages is dependent on interleukin-6. Journal of Infectious Diseases 204, 489–491 (2011). 21742851 10.1093/infdis/jir271

[b15] Tsai T. T. *et al.* An emerging role for the anti-inflammatory cytokine interleukin-10 in dengue virus infection. J Biomed Sci 20, 40, doi: 10.1186/1423-0127-20-40 (2013). 23800014 PMC3700829

[b16] Kou Z. *et al.* Human antibodies against dengue enhance dengue viral infectivity without suppressing type I interferon secretion in primary human monocytes. Virology 410, 240–247, doi: 10.1016/j.virol.2010.11.007 (2011). 21131015

[b17] Jin X., Block O. T., Rose R. & Schlesinger J. Dengue vaccine development and dengue viral neutralization and enhancement assays. Antivir Ther 14, 739–749 (2009). 19812436 10.3851/IMP1288

[b18] Moi M. L., Takasaki T., Saijo M. & Kurane I. Determination of antibody concentration as the main parameter in a dengue virus antibody-dependent enhancement assay using FcgammaR-expressing BHK cells. Archives of virology 159, 103–116, doi: 10.1007/s00705-013-1787-3 (2014). 23900750

[b19] Boonnak K., Slike B. M., Donofrio G. C. & Marovich M. A. Human FcgammaRII cytoplasmic domains differentially influence antibody-mediated dengue virus infection. Journal of immunology 190, 5659–5665, doi: 10.4049/jimmunol.1203052 (2013). PMC3659957 23616574

[b20] Valero N., Espina L. M., Anez G., Torres E. & Mosquera J. A. Short report: increased level of serum nitric oxide in patients with dengue. The American journal of tropical medicine and hygiene 66, 762–764 (2002). 12224588 10.4269/ajtmh.2002.66.762

[b21] Takhampunya R., Padmanabhan R. & Ubol S. Antiviral action of nitric oxide on dengue virus type 2 replication. The Journal of general virology 87, 3003–3011, doi: 10.1099/vir.0.81880-0 (2006). 16963759

[b22] Diaz M. O. *et al.* Homozygous deletion of the alpha-and beta 1-interferon genes in human leukemia and derived cell lines. Proceedings of the National Academy of Sciences 85, 5259–5263 (1988). 10.1073/pnas.85.14.5259 PMC281729 3134658

[b23] Yoneyama M., Onomoto K., Jogi M., Akaboshi T. & Fujita T. Viral RNA detection by RIG-I-like receptors. Current opinion in immunology 32, 48–53 (2015). 25594890 10.1016/j.coi.2014.12.012

[b24] Halstead S. B., Mahalingam S., Marovich M. A., Ubol S. & Mosser D. M. Intrinsic antibody-dependent enhancement of microbial infection in macrophages: disease regulation by immune complexes. The Lancet infectious diseases 10, 712–722 (2010). 20883967 10.1016/S1473-3099(10)70166-3 PMC3057165

[b25] Chan K. R. *et al.* Leukocyte immunoglobulin-like receptor B1 is critical for antibody-dependent dengue. Proceedings of the National Academy of Sciences 111, 2722–2727 (2014). 10.1073/pnas.1317454111 PMC3932915 24550301

[b26] De Vera M. E. *et al.* Transcriptional regulation of human inducible nitric oxide synthase (NOS2) gene by cytokines: initial analysis of the human NOS2 promoter. Proceedings of the National Academy of Sciences 93, 1054–1059 (1996). 10.1073/pnas.93.3.1054 PMC40029 8577713

[b27] Lowenstein C. J. & Padalko E. iNOS (NOS2) at a glance. Journal of cell science 117, 2865–2867 (2004). 15197240 10.1242/jcs.01166

[b28] Hung N. T. *et al.* Dengue hemorrhagic fever in infants: a study of clinical and cytokine profiles. Journal of infectious Diseases 189, 221–232 (2004). 14722886 10.1086/380762

[b29] Richards A. L. & Jackson W. T. How positive-strand RNA viruses benefit from autophagosome maturation. Journal of virology 87, 9966–9972, doi: 10.1128/jvi.00460-13 (2013). 23760248 PMC3754026

[b30] Jounai N. *et al.* The Atg5–Atg12 conjugate associates with innate antiviral immune responses. Proceedings of the National Academy of Sciences 104, 14050–14055 (2007). 10.1073/pnas.0704014104 PMC1955809 17709747

[b31] Zhao Y. *et al.* COX5B regulates MAVS-mediated antiviral signaling through interaction with ATG5 and repressing ROS production. PLoS pathogens 8, e1003086 (2012). 23308066 10.1371/journal.ppat.1003086 PMC3534373

[b32] Mahalingam S. & Lidbury B. A. Suppression of lipopolysaccharide-induced antiviral transcription factor (STAT-1 and NF-κB) complexes by antibody-dependent enhancement of macrophage infection by Ross River virus. Proceedings of the National Academy of Sciences 99, 13819–13824 (2002). 10.1073/pnas.202415999 PMC129781 12364588

[b33] Schoggins J. W. *et al.* A diverse range of gene products are effectors of the type I interferon antiviral response. Nature 472, 481–485 (2011). 21478870 10.1038/nature09907 PMC3409588

[b34] Pulit-Penaloza J. A., Scherbik S. V. & Brinton M. A. Type 1 IFN-independent activation of a subset of interferon stimulated genes in West Nile virus Eg101-infected mouse cells. Virology 425, 82–94 (2012). 22305622 10.1016/j.virol.2012.01.006 PMC3288888

[b35] Olagnier D. *et al.* Inhibition of dengue and chikungunya virus infections by RIG-I-mediated type I interferon-independent stimulation of the innate antiviral response. Journal of virology 88, 4180–4194 (2014). 24478443 10.1128/JVI.03114-13 PMC3993760

[b36] Dixit E. *et al.* Peroxisomes are signaling platforms for antiviral innate immunity. Cell 141, 668–681 (2010). 20451243 10.1016/j.cell.2010.04.018 PMC3670185

[b37] Paladino P., Cummings D. T., Noyce R. S. & Mossman K. L. The IFN-independent response to virus particle entry provides a first line of antiviral defense that is independent of TLRs and retinoic acid-inducible gene I. The Journal of Immunology 177, 8008–8016 (2006). 17114474 10.4049/jimmunol.177.11.8008

[b38] Flipse J., Wilschut J. & Smit J. M. Molecular mechanisms involved in antibody-dependent enhancement of dengue virus infection in humans. Traffic 14, 25–35, doi: 10.1111/tra.12012 (2013). 22998156

[b39] Dhodapkar K. M. *et al.* Selective blockade of the inhibitory Fcγ receptor (FcγRIIB) in human dendritic cells and monocytes induces a type I interferon response program. The Journal of experimental medicine 204, 1359–1369 (2007). 17502666 10.1084/jem.20062545 PMC2118610

[b40] Khor C. C. *et al.* Genome-wide association study identifies susceptibility loci for dengue shock syndrome at MICB and PLCE1. Nature genetics 43, 1139–1141 (2011). 22001756 10.1038/ng.960 PMC3223402

[b41] Callaway J. B. *et al.* Spleen Tyrosine Kinase (Syk) Mediates IL-1β Induction by Primary Human Monocytes During Antibody-Enhanced Dengue Virus Infection. Journal of Biological Chemistry 290, 17306–17320 (2015). 26032420 10.1074/jbc.M115.664136 PMC4498069

[b42] Chen L. C. *et al.* Correlation of serum levels of macrophage migration inhibitory factor with disease severity and clinical outcome in dengue patients. The American journal of tropical medicine and hygiene 74, 142–147 (2006). 16407359

[b43] Libraty D. H. *et al.* Differing influences of virus burden and immune activation on disease severity in secondary dengue-3 virus infections. Journal of Infectious Diseases 185, 1213–1221 (2002). 12001037 10.1086/340365

[b44] Malavige G. N. *et al.* Suppression of virus specific immune responses by IL-10 in acute dengue infection. PLoS neglected tropical diseases 7, e2409, doi: 10.1371/journal.pntd.0002409 (2013). 24040431 PMC3764236

[b45] Tsai T. T. *et al.* Antibody-dependent enhancement infection facilitates dengue virus-regulated signaling of IL-10 production in monocytes. PLoS neglected tropical diseases 8, e3320, doi: 10.1371/journal.pntd.0003320 (2014). 25412261 PMC4239119

[b46] Yoneyama M. *et al.* The RNA helicase RIG-I has an essential function in double-stranded RNA-induced innate antiviral responses. Nature immunology 5, 730–737, doi: 10.1038/ni1087 (2004). 15208624

[b47] Jones M. *et al.* Dengue virus inhibits alpha interferon signaling by reducing STAT2 expression. Journal of virology 79, 5414–5420, doi: 10.1128/JVI.79.9.5414-5420.2005 (2005). 15827155 PMC1082737

[b48] Muñoz-Jordán J. L. *et al.* Inhibition of alpha/beta interferon signaling by the NS4B protein of flaviviruses. Journal of virology 79, 8004–8013 (2005). 15956546 10.1128/JVI.79.13.8004-8013.2005 PMC1143737

[b49] Muñoz-Jordán J. L., Sánchez-Burgos G. G., Laurent-Rolle M. & García-Sastre A. Inhibition of interferon signaling by dengue virus. Proceedings of the National Academy of Sciences 100, 14333–14338 (2003). 10.1073/pnas.2335168100 PMC283592 14612562

[b50] Aguirre S. *et al.* DENV inhibits type I IFN production in infected cells by cleaving human STING. PLoS pathogens 8, e1002934 (2012). 23055924 10.1371/journal.ppat.1002934 PMC3464218

[b51] Jackson W. T. Viruses and the autophagy pathway. Virology 479, 450–456 (2015). 25858140 10.1016/j.virol.2015.03.042 PMC5917100

[b52] Khakpoor A., Panyasrivanit M., Wikan N. & Smith D. R. A role for autophagolysosomes in dengue virus 3 production in HepG2 cells. Journal of General Virology 90, 1093–1103 (2009). 19264601 10.1099/vir.0.007914-0

[b53] Mateo R. *et al.* Inhibition of cellular autophagy deranges dengue virion maturation. Journal of virology 87, 1312–1321 (2013). 23175363 10.1128/JVI.02177-12 PMC3554187

[b54] Panyasrivanit M. *et al.* Induced autophagy reduces virus output in dengue infected monocytic cells. Virology 418, 74–84 (2011). 21813150 10.1016/j.virol.2011.07.010

[b55] Panyasrivanit M., Khakpoor A., Wikan N. & Smith D. R. Co-localization of constituents of the dengue virus translation and replication machinery with amphisomes. Journal of General Virology 90, 448–456 (2009). 19141455 10.1099/vir.0.005355-0

[b56] Jackson W. T. *et al.* Subversion of cellular autophagosomal machinery by RNA viruses. PLoS biology 3, e156 (2005). 15884975 10.1371/journal.pbio.0030156 PMC1084330

[b57] Yorimitsu T. & Klionsky D. J. Endoplasmic reticulum stress:a new pathway to induce autophagy. Autophagy 3, 160–162 (2007). 17204854 10.4161/auto.3653

[b58] Tal M. C. *et al.* Absence of autophagy results in reactive oxygen species-dependent amplification of RLR signaling. Proceedings of the National Academy of Sciences of the United States of America 106, 2770–2775, doi: 10.1073/pnas.0807694106 (2009). 19196953 PMC2650341

[b59] Huang J. *et al.* Activation of antibacterial autophagy by NADPH oxidases. Proceedings of the National Academy of Sciences 106, 6226–6231 (2009). 10.1073/pnas.0811045106 PMC2664152 19339495

[b60] Brooks C. R. *et al.* KIM−1−/TIM−1− mediated phagocytosis links ATG5‐/ULK1‐dependent clearance of apoptotic cells to antigen presentation. The EMBO journal e201489838 (2015). 10.15252/embj.201489838 PMC4601664 26282792

