# Supplementary material for: Antibody-dependent enhancement of dengue virus infection inhibits RLR-mediated Type-I IFN-independent signalling through upregulation of cellular autophagy
Source: Sci Rep. 2016 Feb 29;6:22303. doi: 10.1038/srep22303 (PMC4770412; doi:10.1038/srep22303)
Supplement: Supplementary Information [file srep22303-s1.doc]

**Antibody-dependent enhancement of dengue virus infection inhibits RLR-mediated Type-I IFN-independent signalling through upregulation of cellular autophagy**

Xinwei Huang^1,2^, Yaofei Yue^1,2^, Duo Li^1,2^, Yujiao Zhao^1,2^, Lijuan Qiu^1,2^, Junying Chen^1,2^, Yue Pan^1,2^, Juemin Xi^1,2^, Xiaodan Wang^1,2^, Qiangming Sun ^1,2^*, Qihan Li^1,2^*

*^1^Institute of Medical Biology, Chinese Academy of Medical Sciences, and Peking Union Medical College, Kunming 650118, PR China*

*^2^Yunnan Key Laboratory of Vaccine Research & Development on Severe Infectious Diseases, Kunming 650118, PR China*

***Corresponding author:** Qiangming Sun, **E-mail**: qsun@imbcams.com.cn or Qihan Li, E-mail: [imbcams.lq@gmail.com](mailto:imbcams.lq@gmail.com)

**Current postal address:** Institute of Medical Biology, Chinese Academy of Medical Sciences & Peking Union Medical College (CAMS & PUMC), 935 Jiao Ling Road, Kunming, Yunnan Province 650118, P.R. China

**Telephone number:** 86-871-68335165; **Fax Number:** 86-871-68334483

**Table S1: Primers used for RNA Quantization, plasmids construction or PCR amplification**

**Primer sequence for gene expression quantitation**

| NOS2 | TTCAGTATCACAACCTCAGCAAG |
| --- | --- |
|  | TGGACCTGCAAGTTAAAATCCC |
| RIG-I | CTGGACCCTACCTACATCCTG |
|  | GGCATCCAAAAAGCCACGG |
| MDA5 | GCCCGCTACATGAACCCTG |
|  | CAGCAATCCGGTTTCTGTCTT |
| IRF-1 | CTGTGCGAGTGTACCGGATG |
|  | ATCCCCACATGACTTCCTCTT |
| SOCS3 | CCTGCGCCTCAAGACCTTC |
|  | GTCACTGCGCTCCAGTAGAA |
| IL-6 | CATCCTCGACGGCATCTCAG |
|  | GCTCTGTTGCCTGGTCCTC |
| IL-10 | TCAGGGTGGCGACTCTAT |
|  | TGGGCTTCTTCTAAATCGTTC |
| GAPDH | TCGACAGTCAGCCGCATCT |
|  | CCGTTGACTCCGACCTTCA |

**Primer sequence for gene expression vectors construction**

| RIG-I | AACTCGAGATGACCACCGAGCAGCGACG |
| --- | --- |
|  | AAGTCGACTCATTTGGACATTTCTGCTGGA |
| MDA5 | AACTCGAGATGTCGAATGGGTATTCCAC |
|  | ATGTCGACCTAATCCTCATCACTAAATA |

**Primer sequence for Crispr-Cas9 vector construction**

| IL-10 1.1 | CACCGTCACCCCAGTCAGGAGGACC |
| --- | --- |
|  | AAACGGTCCTCCTGACTGGGGTGAC |
| IL-10 1.2 | CACCGGCATGTTAGGCAGGTTGCCT |
|  | AAACAGGCAACCTGCCTAACATGCC |
| IL-10 2.1 | CACCGTTTCCCTGCTGCAAGGCATG |
|  | AAACCATGCCTTGCAGCAGGGAAAC |
| IL-10 2.2 | CACCGTCTGGAAACGCTCTAAGCAG |
|  | AAACCTGCTTAGAGCGTTTCCAGAC |
| ATG5 1.1 | CACCGTTTTCTTACAAAAATGGACA |
|  | AAACTGTCCATTTTTGTAAGAAAAC |
| ATG5 1.2 | CACCGGGAGTTAAAGCAGTACATAC |
|  | AAACGTATGTACTGCTTTAACTCCC |
| ATG5 1.3 | CACCGAAAATATCTCTTTCACATTG |
|  | AAACCAATGTGAAAGAGATATTTTC |

**Primer sequence for cellular gene detection**

| IFNA2 | TTTGCTTTACTGGTGGCCCT |
| --- | --- |
|  | AAGGTGAGCTGGCATACGAA |
| IFNB1 | AATTGTCCAGTCCCAGAGGC |
|  | AGTAGGCGACACTGTTCGTG |

**Primer sequence for PCR detection of gene deletion or sequencing**

| IL-10 | AGGTCTACACATCAGGGGCT |
| --- | --- |
|  | CTATGTCAACCCTTCGGGGC |
| ATG5 | GGGGGCCACTTTTGGTAGAG |
|  | AGTCCAGAACGCATCATGACA |

**Supplementary Figure Legends**

**Fig S1. Binding activities of monoclonal antibodies to DENV3.** Commercial monoclonal antibodies 2H2 (MsX Dengue complex, MAB8705), 4G2 (mouse anti-flavivirus group, MAB10216), anti-PrM (anti-dengue virus PrM glycoprotein antibody, AB41473) and Control IgG were tested for Binding activities to DENV3 with ELISA method.

**Fig S2. Deletion of type 1 IFN(IFN α/β) in K562 cells.** THP-1, K562 and U937 genomic DNA was extracted for IFNA2, IFNB1 and IFNG detection. (a)PCR detection of IFNA2, IFNB1, and IFNG gene in THP-1, K562 and U937 with primers shown in table S1. (b) Dot blot of DNA from THP-1, K562 and U937 cells. Genomic DNA were extracted and subjected to dot blot by neutral transfer. Samples of DNA were loaded in three spots for each sample at indicated amount. The probe was labeled with biotin by random primer method in the presence of Klenow fragment.

**Fig S3. Treatment of inhibitors of NOS2 (SMT) does not induce cytotoxicity in DENV infected or DENV-ADE infected K562.** K562 cells was pretreated with SMT for 1 h, and then infected with DENV3 with or without enhancing antibodies for 48 h. Purple formazan was used to indicate cell viability. The relative data, as compared with control(no drug), shown represent mean ± SEM values of three independent experiments. NS, not significant.

**Fig S4. Treatment of rapamycine and 3-MA does not induce cytotoxicity in DENV infected or DENV-ADE infected K562.** K562 cells was pretreated with rapamycine(a) or 3-MA(b) for 1 h, and then infected with DENV3 with or without enhancing antibodies for 48 h. Purple formazan was used to indicate cell viability. The relative data, as compared with control(no drug), shown represent mean ± SEM values of three independent experiments. NS, not significant.

**Fig S5: Sequencing validation of K562 isolates carrying homozygous deletion in IL-10 and ATG5 locus.** (a) Sequencing of IL-10 locus in widetype K562. (b) sequencing of IL-10 locus in K562(sg1.1+2.1)isolates. (c) sequencing of IL-10 locus in K562(sg1.1+2.2)isolates. (d) sequencing of IL-10 locus in K562(sg1.2+2.1)isolates. (e) sequencing of IL-10 locus in K562(sg1.2+2.2)isolates. (f) sequencing of ATG5 locus in widetype K562. (g) sequencing of ATG5 locus in K562(sg1.1+1.3)isolates. (h) sequencing of ATG5 locus in K562(sg1.1+1.2)isolates.

**
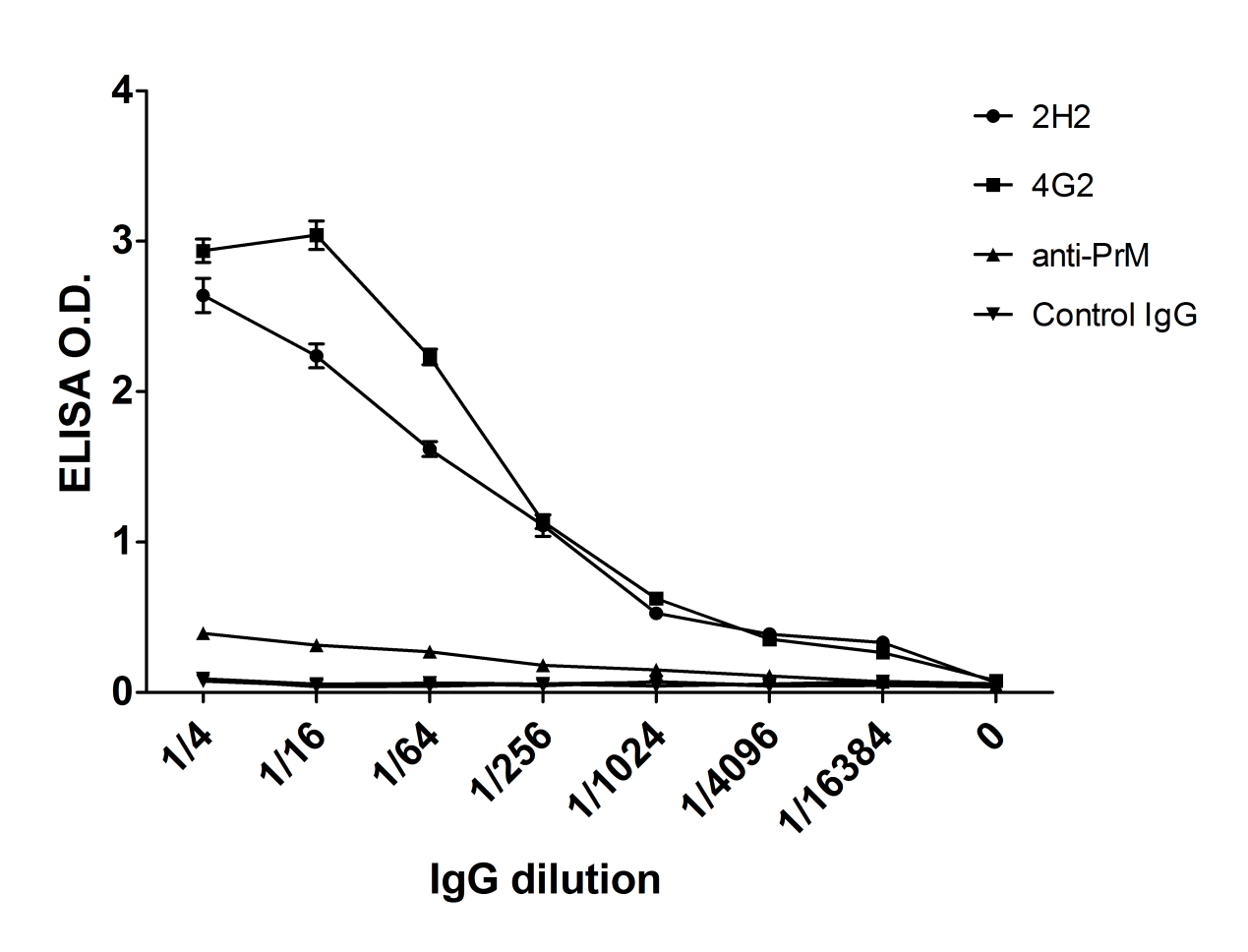
**

**Fig S1**

**
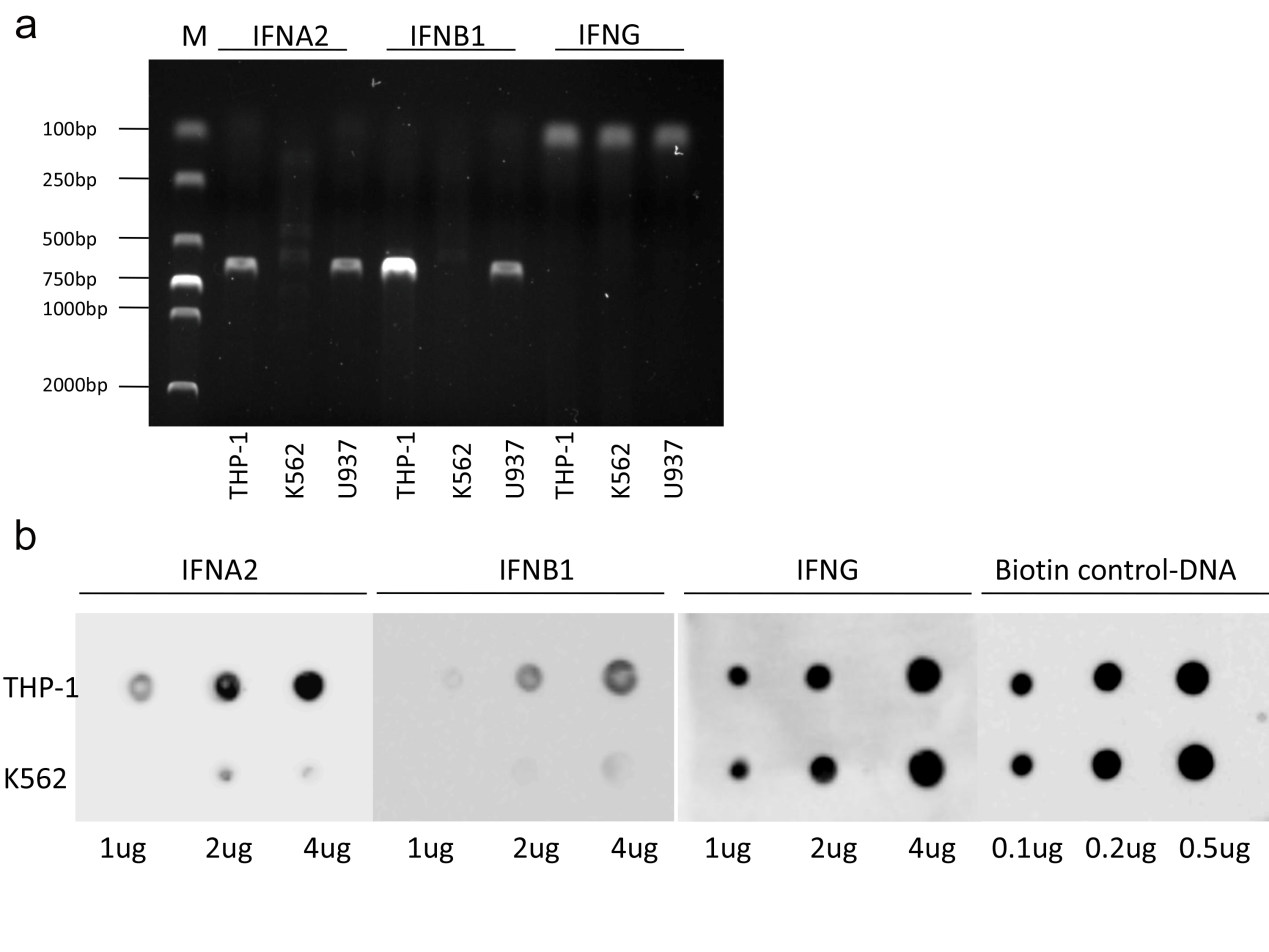
**

**Fig S2**

**
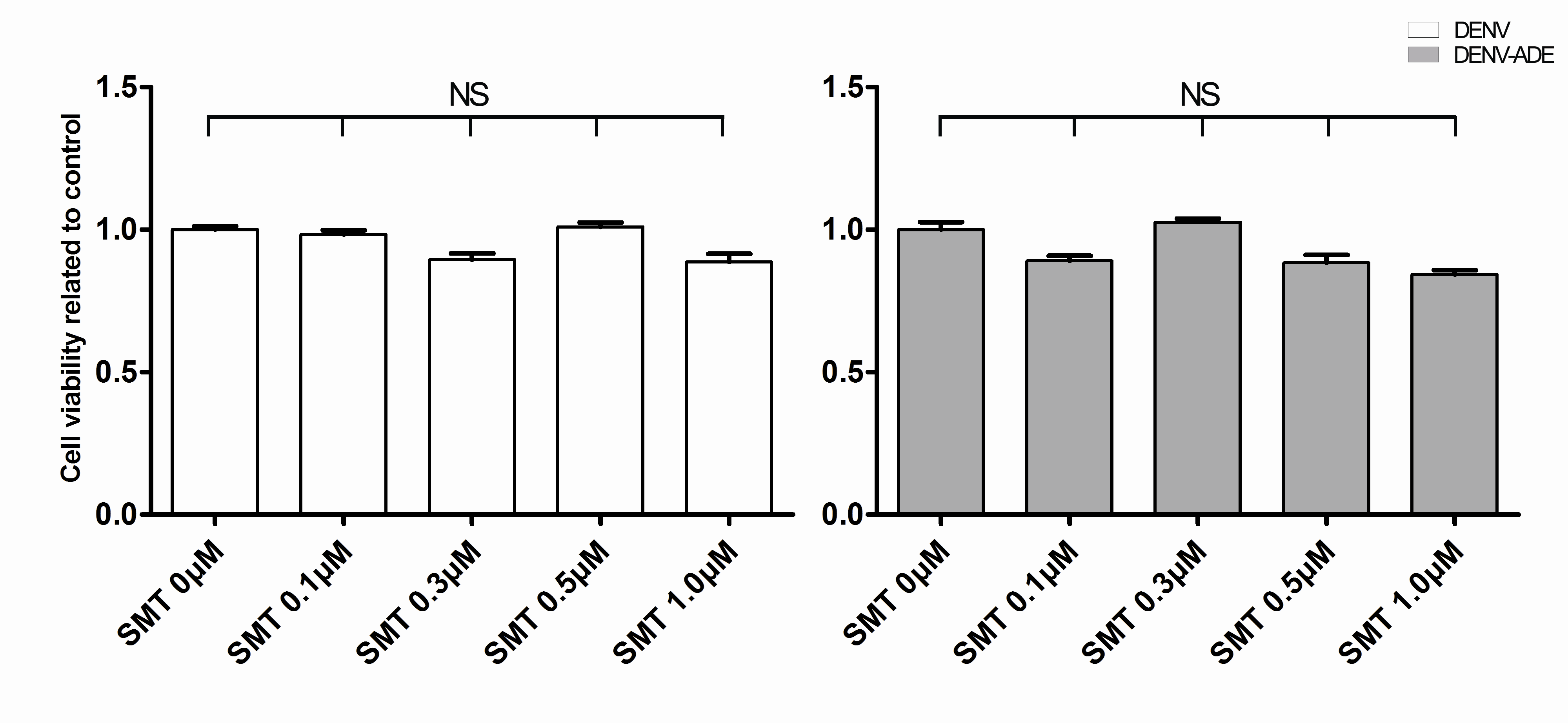
**

**Fig S3**

**
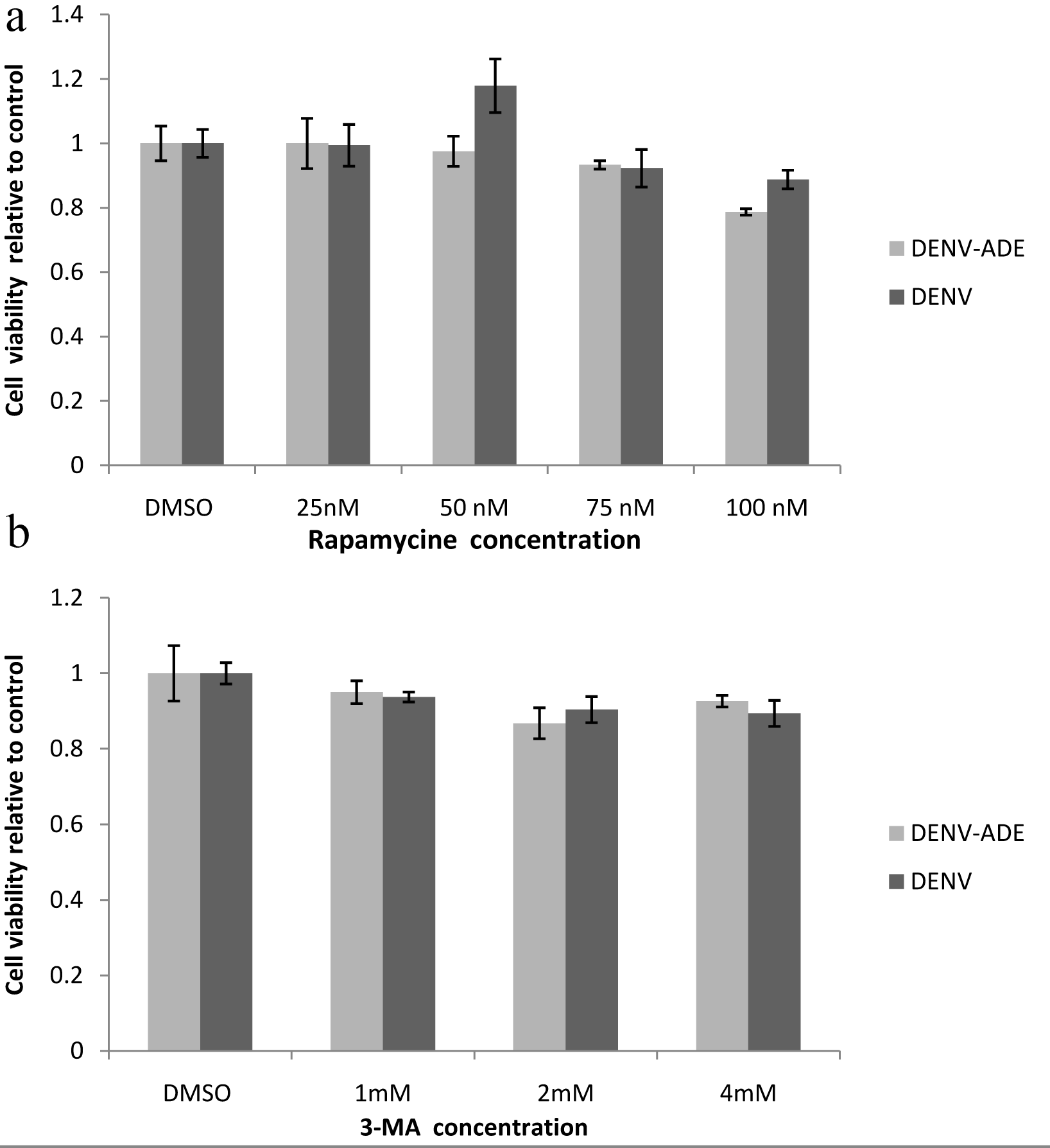
**

**Fig S4**


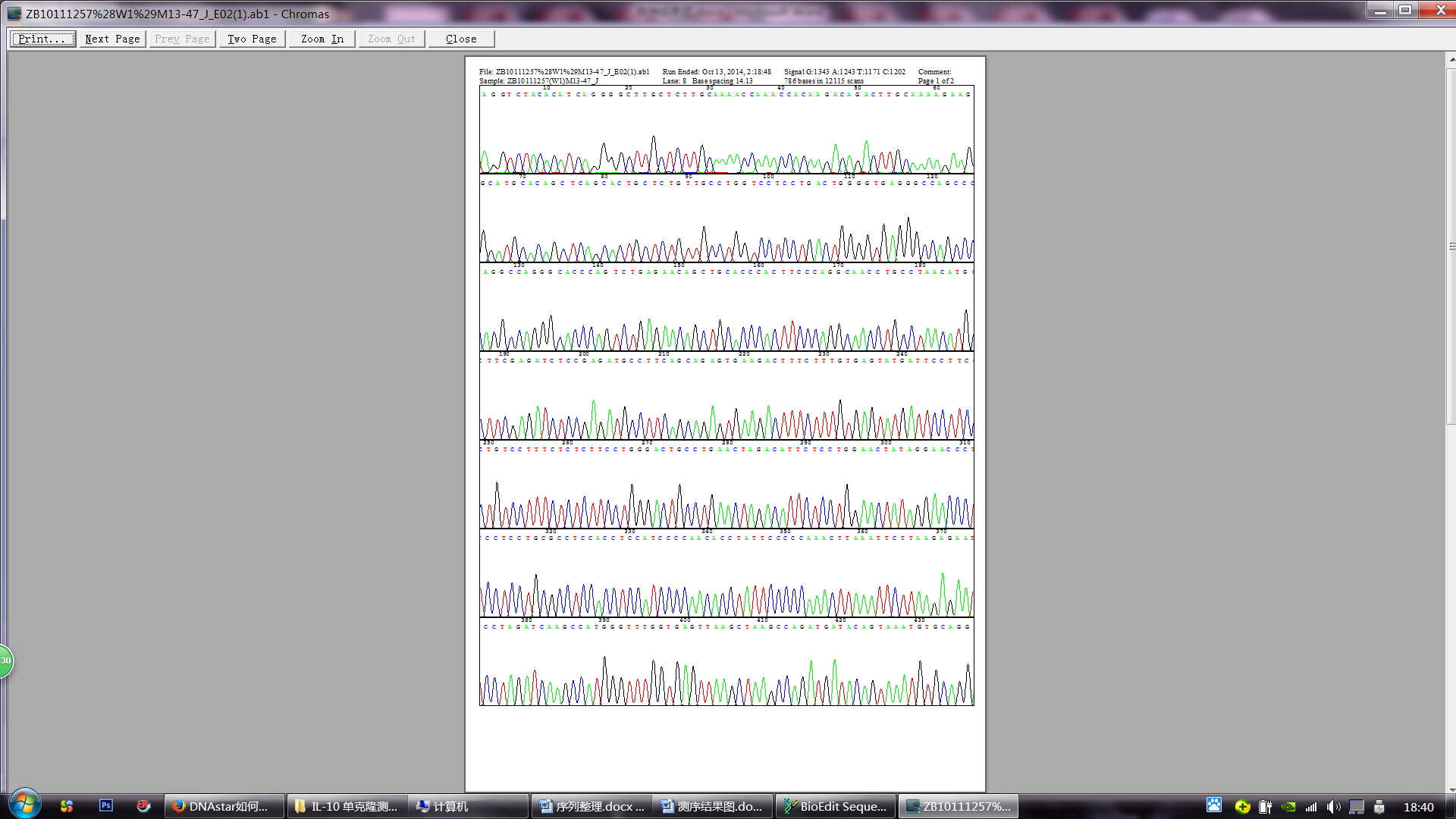


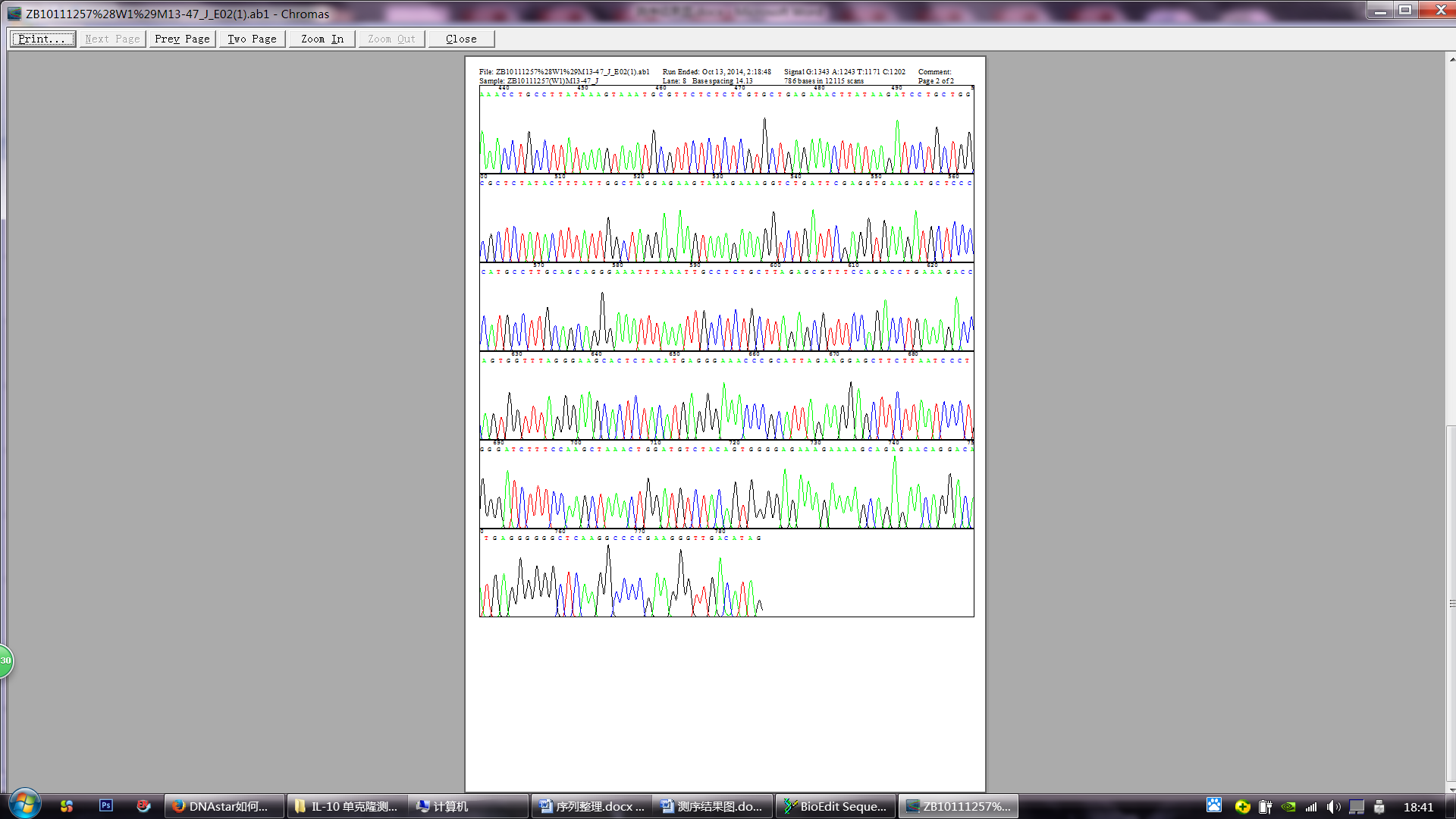


**Fig S5a. sequencing of IL-10 locus in widetype K562**


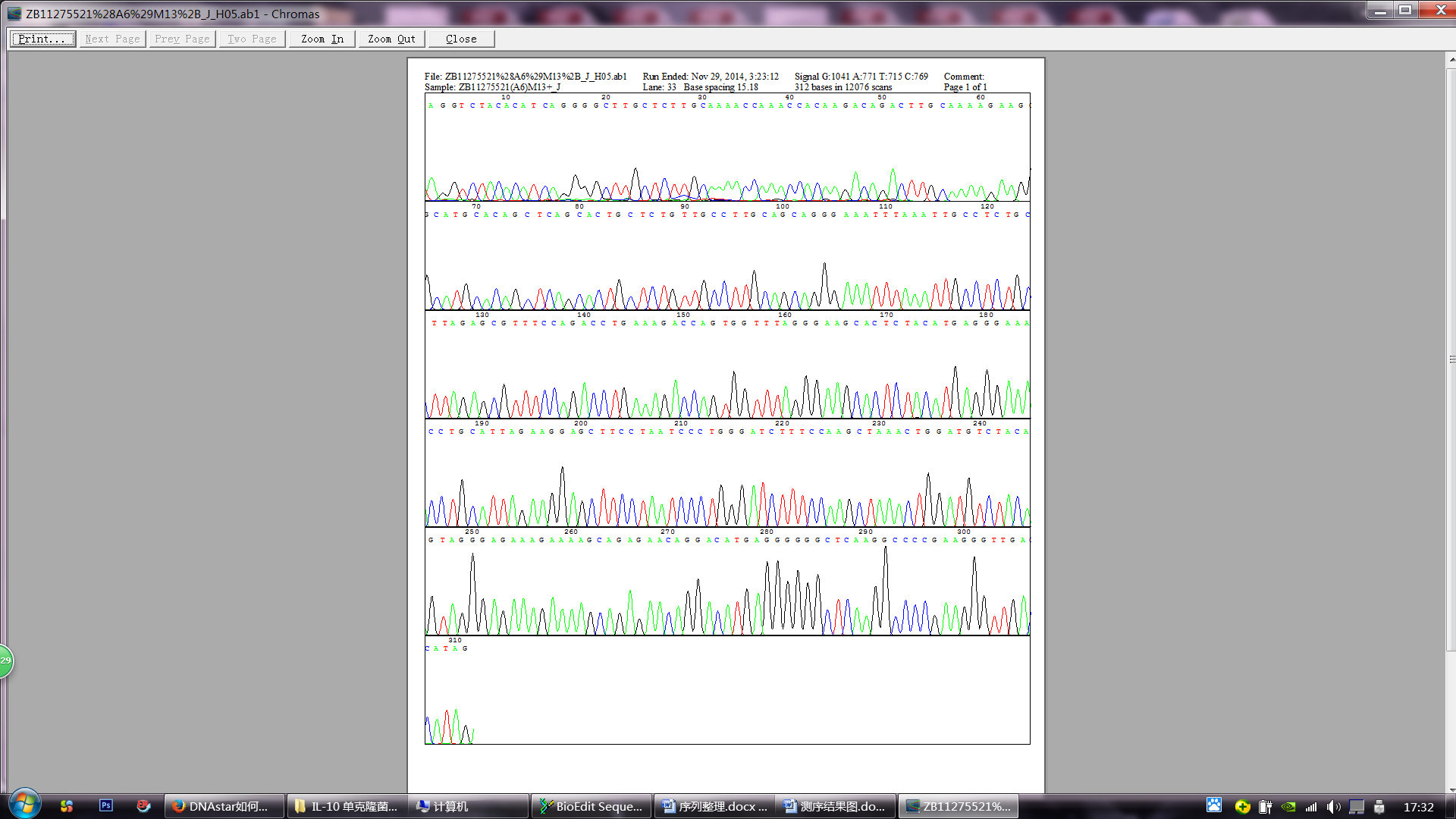


**Fig S5b. sequencing of IL-10 locus in K562（sg1.1+2.1）isolates**


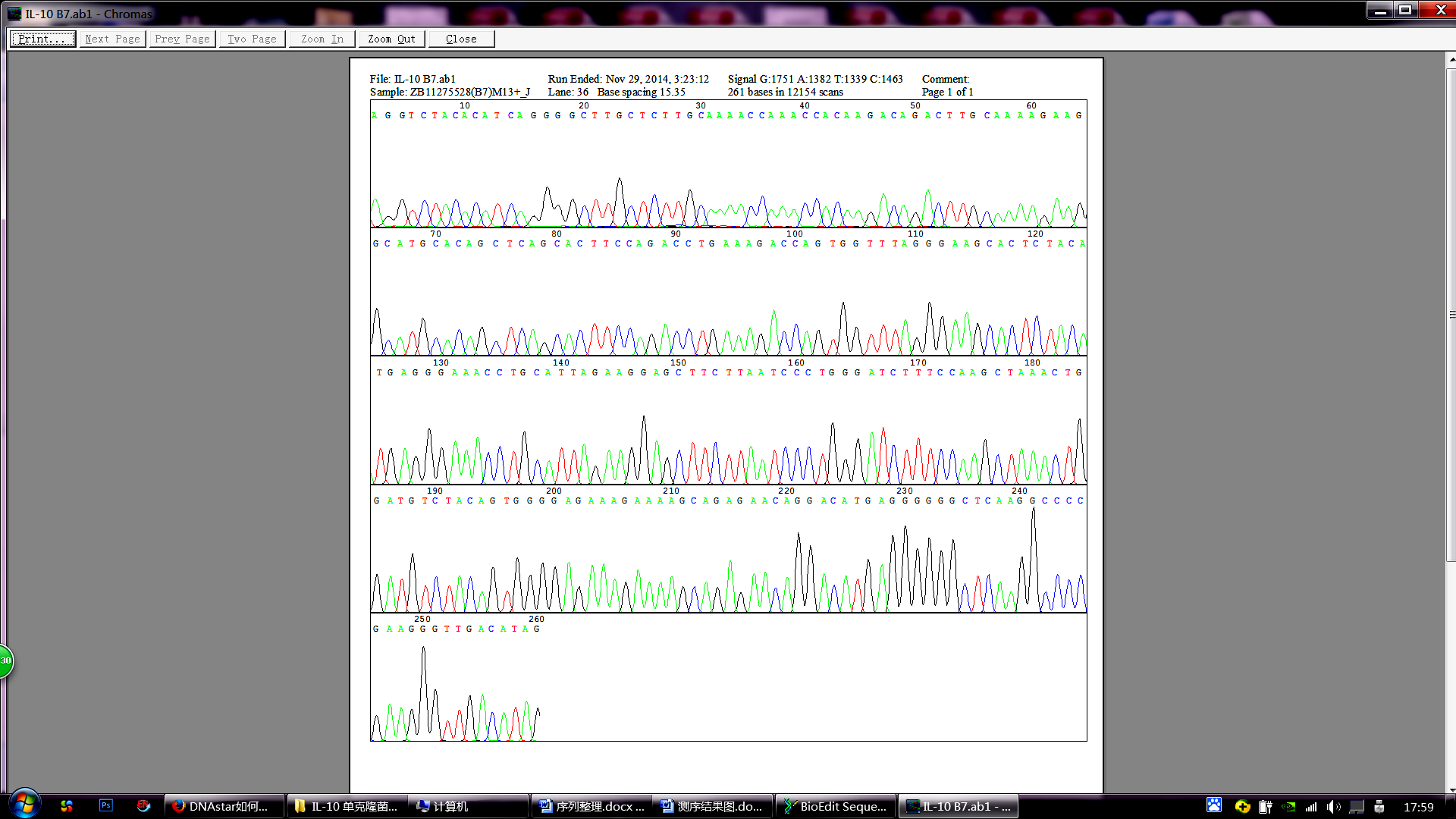


**Fig S5c. sequencing of IL-10 locus in K562（sg1.1+2.2）isolates**


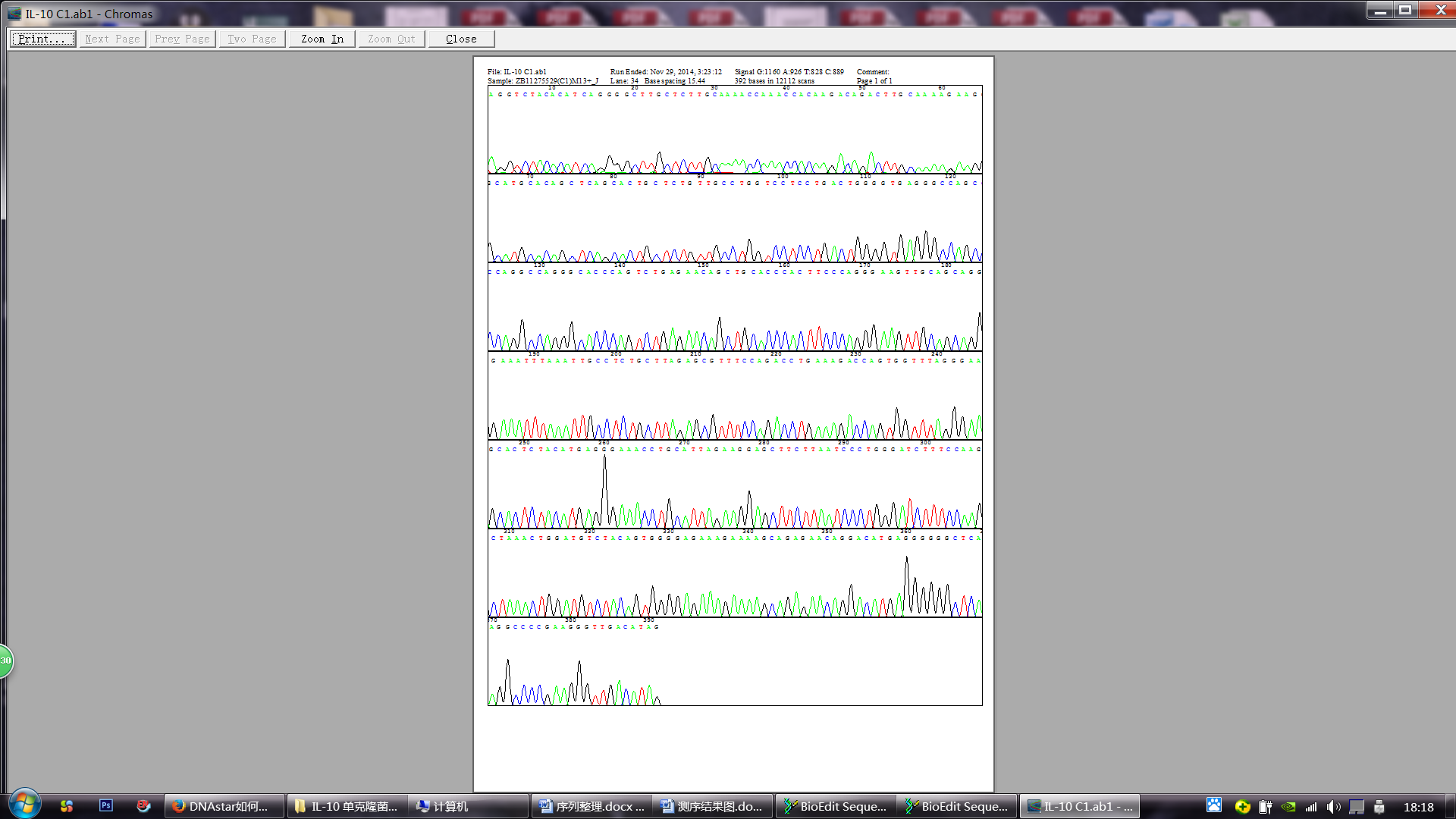


**Fig S5d. sequencing of IL-10 locus in K562（sg1.2+2.1）isolates**


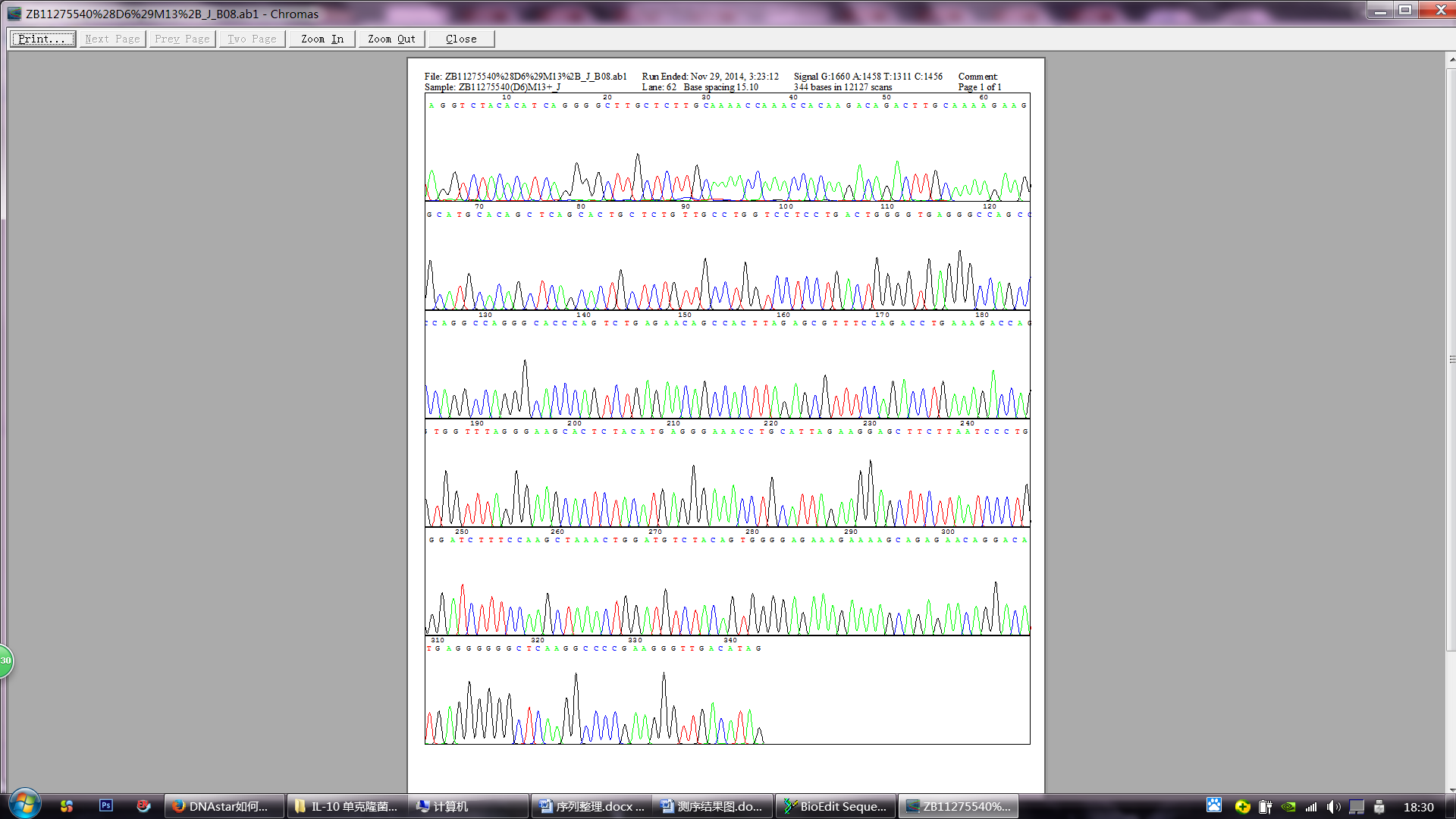


**Fig S5e. sequencing of IL-10 locus in K562（sg1.2+2.2）isolates**


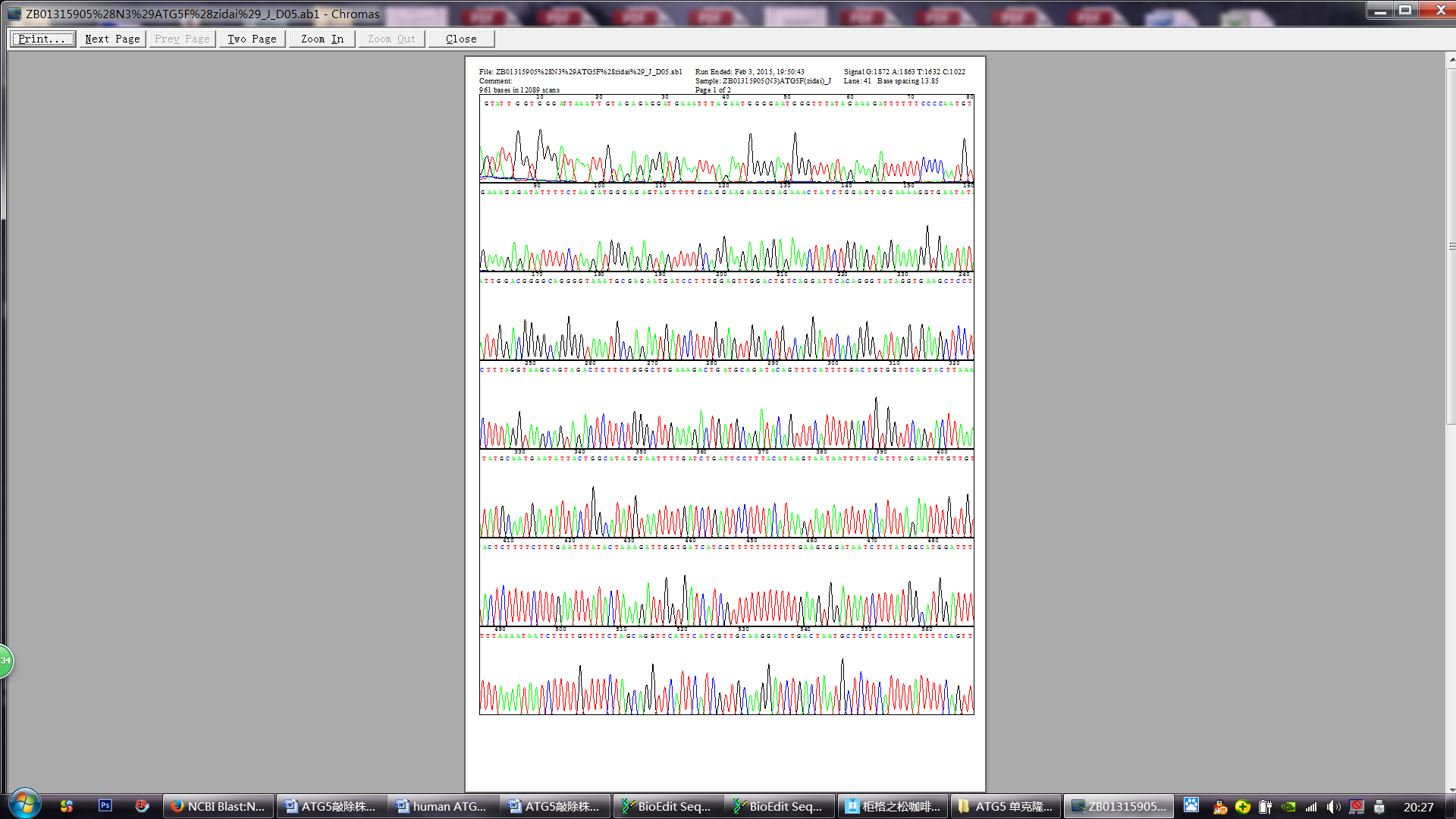


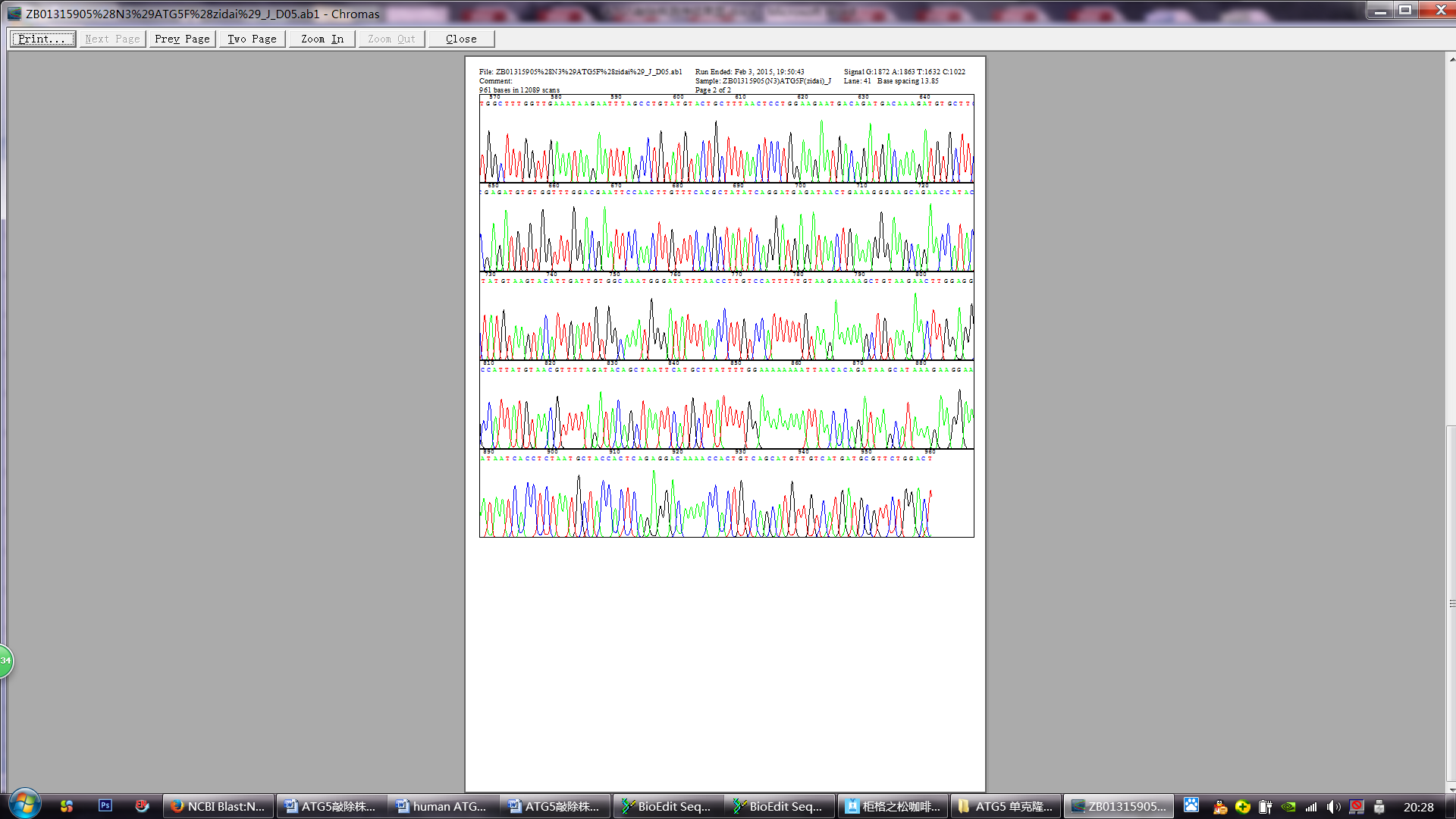


**Fig S5f. sequencing of ATG5 locus in widetype K562**


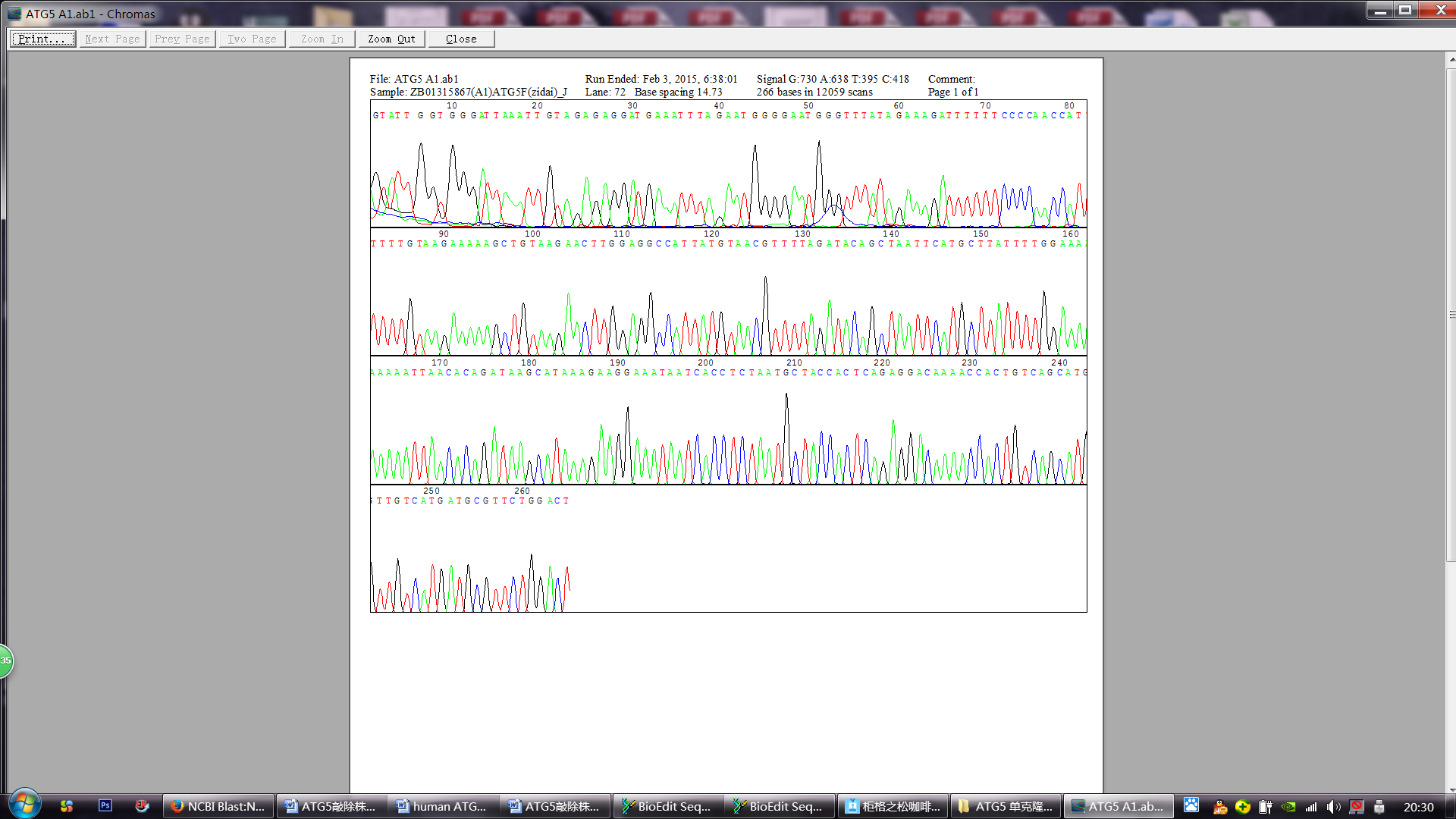


**Fig S5g. sequencing of ATG5 locus in K562（sg1.1+1.3）isolates**


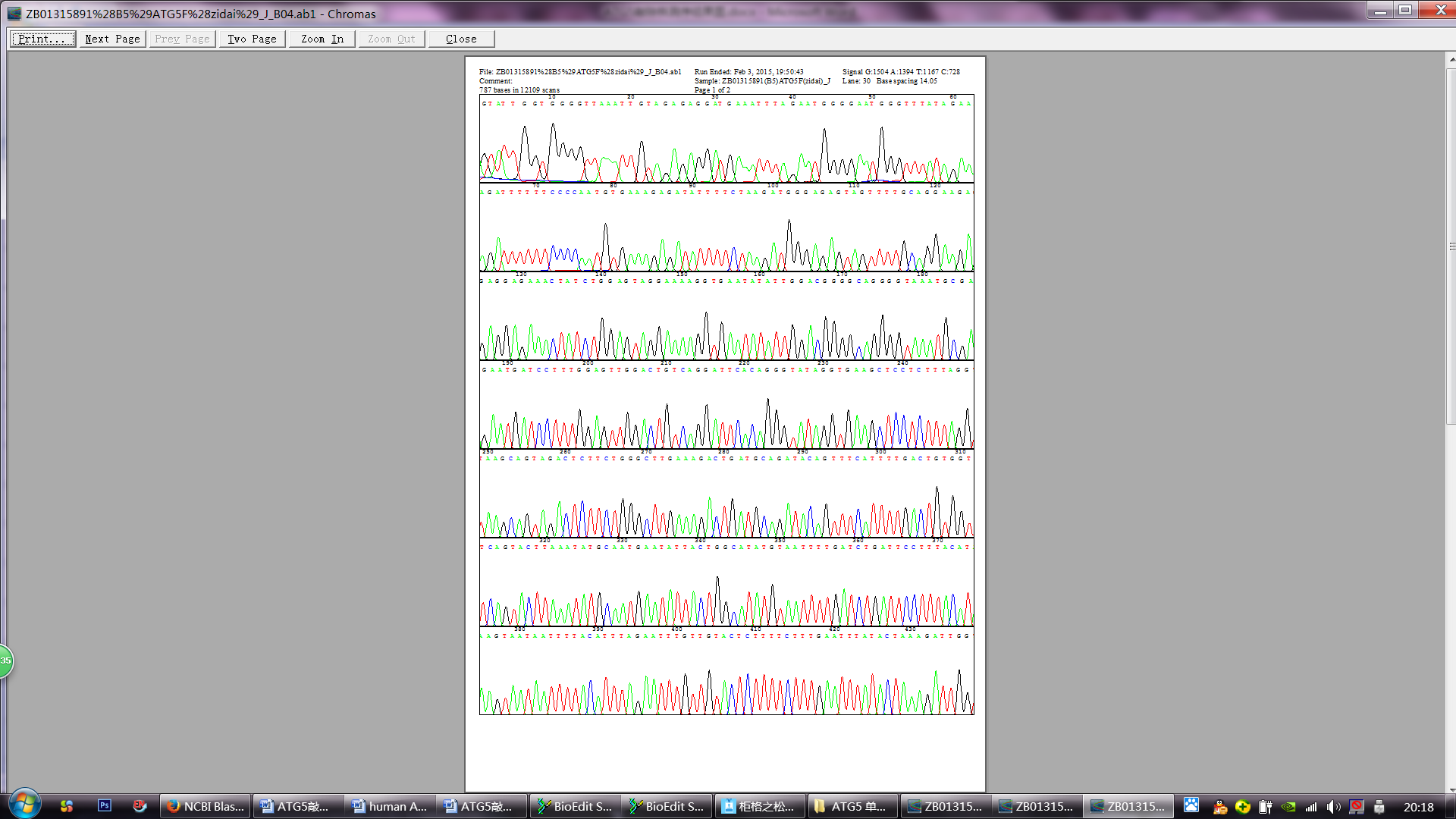


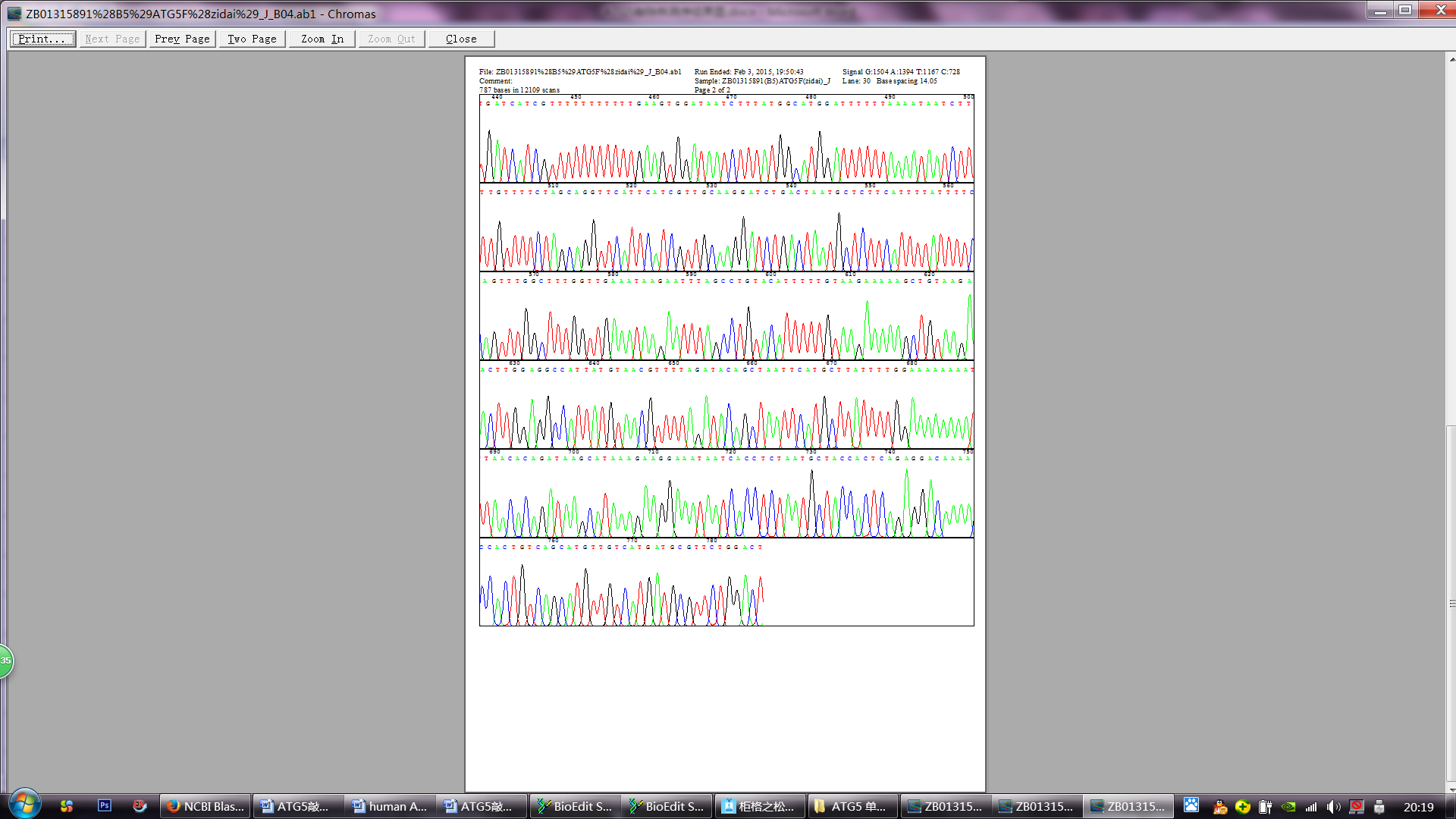


**Fig S5h. sequencing of ATG5 locus in K562（sg1.1+1.2）isolates**
